# Identification of Brain Cell Type‐Specific Therapeutic Targets for Glioma From Genetics

**DOI:** 10.1111/cns.70185

**Published:** 2024-12-25

**Authors:** Jiawei Gui, Jiali Chen, Keqi Wan, Ying Liu, Kai Huang, Xingen Zhu

**Affiliations:** ^1^ The 2nd Affiliated Hospital, Jiangxi Medical College, Nanchang University Nanchang Jiangxi China; ^2^ Jiangxi Province Key Laboratory of Neurological Diseases Nanchang Jiangxi China; ^3^ JXHC Key Laboratory of Neurological Medicine Nanchang Jiangxi China; ^4^ Institute of Neuroscience, Jiangxi Medical College, Nanchang University Nanchang Jiangxi China; ^5^ HuanKui Academy, Jiangxi Medical College, Nanchang University Nanchang China; ^6^ The First Clinical Medical College, Jiangxi Medical College, Nanchang University Nanchang Jiangxi China; ^7^ The MOE Basic Research and Innovation Center for the Targeted Therapeutics of Solid Tumors Jiangxi Medical College, Nanchang University Nanchang Jiangxi China

**Keywords:** Bayesian colocalization, brain cells, glioma, Mendelian randomization, therapeutic target

## Abstract

**Background:**

Previous research has demonstrated correlations between the complex types and functions of brain cells and the etiology of glioma. However, the causal relationship between gene expression regulation in specific brain cell types and glioma risk, along with its therapeutic implications, remains underexplored.

**Methods:**

Utilizing brain cell type‐specific cis‐expression quantitative trait loci (cis‐eQTLs) and glioma genome‐wide association study (GWAS) datasets in conjunction with Mendelian randomization (MR) and colocalization analyses, we conducted a systematic investigation to determine whether an association exists between the gene expression of specific brain cell types and the susceptibility to glioma, including its subtypes. Additionally, the potential pathogenicity was explored utilizing mediation and bioinformatics analyses. This exploration ultimately led to the identification of a series of brain cell‐specific therapeutic targets.

**Results:**

A total of 110 statistically significant and robust associations were identified through MR analysis, with most genes exhibiting causal effects exclusively in specific brain cell types or glioma subtypes. Bayesian colocalization analysis validated 36 associations involving 26 genes as potential brain cell‐specific therapeutic targets. Mediation analysis revealed genes indirectly influencing glioma risk via telomere length. Bioinformatics analysis highlighted the involvement of these genes in glioma pathogenesis pathways and supported their enrichment in specific brain cell types.

**Conclusions:**

This study, employing an integrated approach, demonstrated the genetic susceptibility between brain cell‐specific gene expression and the risk of glioma and its subtypes. Its findings offer novel insights into glioma etiology and underscore potential therapeutic targets specific to brain cell types.

## Graphical abstract

By integrating brain cell type‐specific cis‐eQTLs with glioma GWAS dataset using MR and Bayesian colocalization analyses, we elucidated genes with causal effects in distinct brain cell types or glioma subtypes and pinpointed potential brain cell type‐specific therapeutic targets.

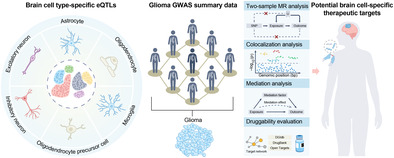

**Abbreviations**
cis‐eQTLs: cis‐expression quantitative trait loci
CMap: Connectivity Map
CNS: central nervous system
CNV: copy number variation
CTPs: cell‐type proportions
DLPFC: dorsolateral prefrontal cortex
FDR: false discovery rate
GBM: glioblastoma
GO: Gene Ontology
GWAS: genome‐wide association study
IV: instrumental variable
IVW: inverse variance weighted
KEGG: Kyoto Encyclopedia of Genes and Genomes
LD: linkage disequilibrium
LDLc: low‐density lipoprotein cholesterol levels
MR: Mendelian randomization
OPCs: oligodendrocyte progenitor cells
PPI: protein–protein interaction
SNPs: single nucleotide polymorphisms
snRNA‐seq: single‐nucleus RNA sequencing
SNV: single nucleotide variant
TKIs: tyrosine kinase inhibitors

## 1 Background

Gliomas, the most prevalent primary intracranial tumors within the central nervous system (CNS), can be generally categorized into glioblastoma (GBM) and lower‐grade non‐GBM types [1]. Among these, GBM (CNS WHO grade 4) is the most aggressive, being a highly malignant tumor characterized by rapid progression, a high recurrence rate, and a dismal prognosis, with a median overall survival time of merely 16–18 months [2]. The clinical management of gliomas, particularly GBM, continues to pose a formidable challenge owing to the scarcity of efficacious therapeutic modalities. Furthermore, the definitive etiology of glioma development remains elusive, and specific studies have indicated an association between exposure to moderate or high levels of ionizing radiation and the risk of developing gliomas, albeit in a minority of instances [3, 4].

The neurological modulation of cancer (known as cancer neuroscience) is an emerging discipline focusing on defining the interactions between the nervous system and cancer and targeting them therapeutically [5, 6]. Various preclinical models have indicated that nervous system activity and neural mechanisms play regulatory roles in glioma development, significantly impacting glioma progression, invasion, and metastasis [7, 8, 9, 10]. For example, the intrinsic characteristics of oligodendrocyte progenitor cells (OPCs) render them highly vulnerable to oncogenic mutations. When coupled with the appropriate combinations of oncogene expression and tumor suppressor loss, OPCs can revert to a highly proliferative state, thereby serving as the cellular origins of gliomas [7, 8]. Neuronal activity has been shown to modulate glioma formation and promote glioma proliferation and growth [9, 10]. These novel insights have prompted researchers to concentrate on elucidating the intricate interactions between the nervous system and gliomas, with a particular emphasis on the intricate types and functions of brain cells, as well as their associations with the etiology of gliomas, ultimately facilitating the discovery of novel therapeutic targets.

Genome‐wide association studies (GWASs) have bolstered the genetic etiology of gliomas, thereby revolutionizing our comprehension of their heritability [11, 12, 13, 14]. The use of Mendelian randomization (MR) analysis of genetic variants in gliomas to ascertain causal factors and, consequently, unravel the mechanisms underlying glioma initiation has emerged as a prevalent methodology, evolving into a pivotal instrument for drug development [15, 16, 17]. Furthermore, the rapid progression of single‐cell genomics currently offers a formidable method to elucidating how genetic variations at the brain cellular level modulate gene expression through the identification of brain cell type‐specific eQTLs and their integration with GWAS data, thereby uncovering brain cell‐specific pathological consequences [18, 19]. Consequently, this avenue presents a highly promising trajectory for investigating the neurological origins of gliomas and discovering novel cancer neuroscience‐driven therapeutic targets for gliomas.

In this study, we focused on the genetic regulation of gene expression at the cellular level, utilizing data from six major brain cell type‐specific cis‐eQTLs to identify causal genes affecting glioma susceptibility in specific brain cell types through two‐sample MR analysis. We subsequently performed colocalization, mediation, and bioinformatics analyses to screen for these causal genes, assess their potential as brain cell type‐specific therapeutic targets, and explore potential pathogenic mechanisms. In addition, based on the strategy of gene expression perturbation, we predicted several potential target drugs to facilitate clinical drug development.

## 2 Methods

**FIGURE 1:**
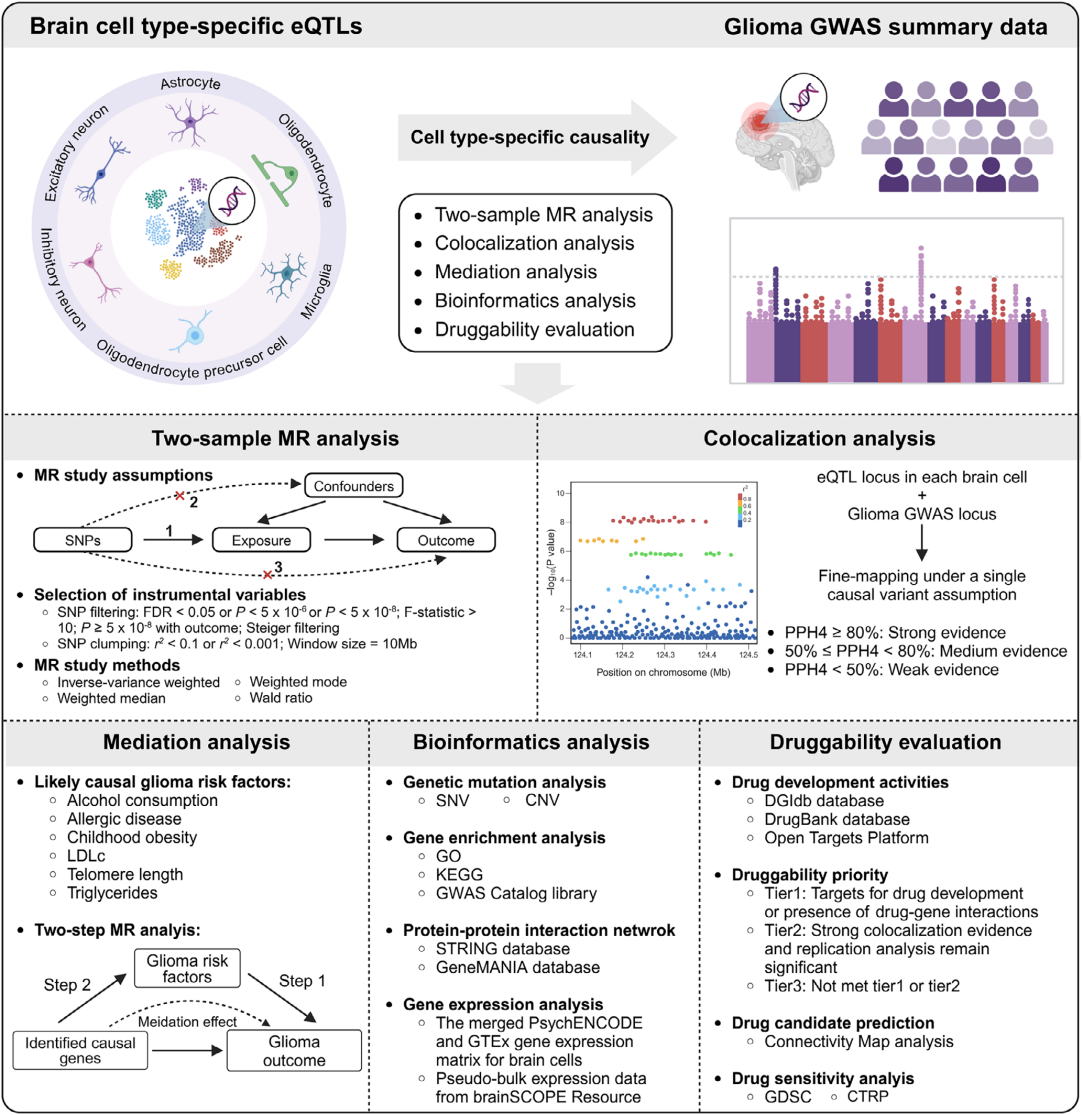
Overview of the study design. The three core assumptions of MR studies are as follows: (1) association with the exposure, (2) independence from confounders, and (3) influence on the outcome exclusively through the exposure. The figure was created with BioRender.com.

The flowchart of the overall study design is shown in Figure 1. Further details of the methods are provided as follows.

### 2.1 Data Sources

Brain cell type‐specific cis‐eQTLs derived from published studies [18, 19]. Fujita et al. analyzed single‐nucleus RNA sequencing (snRNA‐seq) data from the dorsolateral prefrontal cortex (DLPFC) of 424 individuals to identify cis‐eQTLs for seven brain cell types. Bryois et al. identified cis‐eQTLs using snRNA‐seq from 373 brain samples of eight brain cell types derived from the prefrontal cortex, temporal cortex, and white matter. Here, we collected cis‐eQTLs for six major brain cell types (astrocytes, inhibitory neurons, excitatory neurons, microglia, oligodendrocytes, and OPCs, respectively) for subsequent analyses. Cis‐eQTLs for brain tissue and gene expression matrix for brain cells were obtained from an integrative analysis of the human brain by the PsychENCODE Consortium [20]. The cis‐eQTLs were calculated on 1387 filtered adult samples (set with a false discovery rate (FDR) < 0.05 and a filter requiring genes to have an expression > 0.1 FPKM in at least 10 samples) with matching gene expression and genotypes. The merged PsychENCODE and GTEx gene expression matrix for brain cells was normalized and transformed to log_2_(TPM + 1). Pseudo‐bulk snRNA‐Seq expression data for brain cell types from brainSCOPE Resource (https://brainscope.gersteinlab.org/), listing logCPM normalized expression values for subsets of 388 individuals. The detailed clinical characteristics of these individuals were also obtained. Genetic mutation, gene expression (normalized and transformed to log_2_(TPM + 1)), and clinical information data for glioma samples, including GBM and lower‐grade glioma (LGG), were obtained from the TCGA database.

The GWAS summary statistics of glioma were collected from the largest meta‐analysis to date of eight studies with individuals of European ancestry, which contained 12,488 cases (6183 GBM, 5820 non‐GBM) and 18,169 controls [11]. The GWAS summary statistics for six brain cell‐type proportions (CTPs) were obtained from Yap et al. [21], who developed a brain cell type‐specific reference panel for CTP deconvolution and leveraged genetic data available for 873 participants of European ancestry to perform a GWAS.

The list of likely causal glioma risk factors was derived from several published MR studies [22, 23, 24]. We identified six likely causal glioma risk factors (alcohol consumption, allergic disease, childhood obesity, low‐density lipoprotein cholesterol levels (LDLc), telomere length, and triglycerides, respectively). All GWAS summary statistics of these risk factors are publicly available through the IEU Open GWAS Project database. Detailed information on GWAS datasets used in this study is presented in Table S1.

For each dataset, we employed the Shapiro–Wilk test and Kolmogorov–Smirnov test to assess the normality of the distribution. We utilized parametric tests in subsequent statistical analyses for normally distributed data, whereas non‐parametric equivalent methods were applied to datasets that did not meet the normality assumption.

### 2.2 Selection of Instrumental Variables (IVs) for MR Analysis

We focused only on protein‐coding genes to identify therapeutic targets. Comprehensive gene annotations (gencode.v45.annotation.gtf) were obtained from GENCODE to screen for protein‐coding genes.

A series of screening criteria were established to ensure that IVs satisfy the three core assumptions of MR studies [25]. First, for brain cell type‐specific cis‐eQTLs (within 1 Mb of the transcription start site of each gene), the exposure‐related single nucleotide polymorphisms (SNPs) that passed the FDR threshold (with FDR < 0.05), where the FDR was computed via the Benjamini‐Hochberg method, were selected. Additionally, the linkage disequilibrium (LD) coefficient *r* ^2^ was set to 0.1, and the LD window width was established at 10 Mb to ensure the independence of SNPs. For glioma risk factors, SNPs meeting genome‐wide significance (*p* < 5.00 × 10^−8^) were utilized, with the LD coefficient set to *r* ^2^ < 0.001 within a clumping window of 10 Mb. For brain CTPs, a more relaxed threshold (*p* < 5.00 × 10^−6^) was applied to obtain sufficient SNPs. Furthermore, SNPs with an *F*‐statistic > 10 were selected to ensure adequate statistical strength. Ultimately, to mitigate the potential for reverse causality and ensure compliance with the exclusion restriction assumption, the selected SNPs passed Steiger filtering and were not associated with the outcome (*p* ≥ 5.00 × 10^−8^).

### 2.3 Two‐Sample MR Analysis

After estimates of the association between genetic variation and exposure or outcome were extracted, the direction of the estimates was coordinated according to the effect allele. Palindromic SNPs exhibiting an intermediate allele frequency greater than 0.42 were excluded during this process. For exposures with multiple instrumental variables (IVs), the inverse variance weighted (IVW) method served as the primary MR analysis method. The Wald ratio method was employed for exposures with a single IV. For exposures involving three or more IVs, the results were supplemented with the weighted median and MR‐Egger regression methods. These methodologies rely on distinct assumptions and function as sensitivity analyses, evaluating the robustness of MR analysis outcomes. Notably, MR‐Egger regression specifically assesses directional pleiotropy by analyzing the intercept term [26]. If these methodologies yield directionally consistent estimates, the reliability of the MR analysis results is significant.

Furthermore, Cochran's *Q* and Rucker's *Q* tests were utilized to evaluate heterogeneity among the IVs. In instances where directed pleiotropy is identified, or *p* < 0.05 for tests of Cochran's *Q*, MR‐Egger (when *p* ≥ 0.05 for tests of Rucker's *Q*), or the weighted median method (when *p* < 0.05 for tests of Rucker's *Q*) are regarded as the primary MR approaches. For brain cell type‐specific eQTL MR analysis, MR estimates yielding *p* values below the Bonferroni‐corrected threshold (0.05 divided by the number of genes utilized for MR analysis in a particular cell type) are deemed statistically significant (refer to Table S2). The MR analyses were conducted via the TwoSampleMR package (version 0.6.5) [27] in R version 4.2.1. The plots were produced utilizing a range of R software packages, including ggplot2 (version 3.4.4) and ComplexHeatmap (version 2.12.1).

### 2.4 Bayesian Colocalization Analysis

For associations supported by MR analyses, we employed Bayesian colocalization analysis to ascertain whether the two traits are influenced by identical or distinct causal variants within a specific genomic region, thereby offering insights into the relationship between genetic signals at brain cell type‐specific eQTLs loci and glioma GWAS loci. The analysis yielded five hypotheses: (i) PPH0: Neither trait is associated; (ii) PPH1: Only trait 1 is associated with genetic variation; (iii) PPH2: Only trait 2 is associated with genetic variation; (iv) PPH3: Both traits are associated with genetic variation, albeit due to distinct genetic variants; (v) PPH4: Both traits are associated with genetic variation, and this association stems from the same genetic variation. The priori probabilities were assigned as p1 = 1 × 10^−4^, p2 = 1 × 10^−4^, and p12 = 1 × 10^−5^. Each hypothesis corresponds to a posterior probability. In this study, a posterior probability of PPH4 ≥ 80% signifies strong evidence of colocalization between eQTLs and glioma GWAS. A posterior probability of PPH4 ≥ 50% indicates medium evidence of colocalization between eQTLs and glioma GWAS. Bayesian colocalization analysis was performed via the coloc R package (version 5.2.3).

### 2.5 Mediation Analysis

We conducted a two‐step MR analysis (Step‐1 MR: glioma risk factors → glioma outcomes; Step‐2 MR: genes identified by MR and Bayesian colocalization analyses → glioma risk factors), to elucidate the potential mediating influence of glioma risk factors on the associations between causal genes at the brain cell level and glioma outcomes. To quantify the impact of genes on glioma outcomes mediated through glioma risk factors, we employed the product‐of‐coefficients approach to estimate the strength of the indirect effects, utilizing the Delta method to ascertain the standard error (SE) and confidence interval (CI). This mediation analysis utilized the Mediation R package (Version: 1.2.2).

### 2.6 Bioinformatics Analysis

To investigate the expression patterns of genes identified through MR and Bayesian colocalization analyses across diverse brain cell types and to delve deeper into their potential contributions to glioma pathogenesis, we conducted genetic mutation, gene enrichment, protein–protein interaction (PPI), and gene expression analyses. The Kyoto Encyclopedia of Genes and Genomes (KEGG), GWAS Catalog library, and Gene Ontology (GO) analyses were performed via the Enrichr web tool (https://maayanlab.cloud/Enrichr/). We focused on three GO terms: biological process (BP), molecular function (MF), and cellular component (CC). Additionally, we constructed a PPI network utilizing the STRING database (https://string‐db.org) and the GeneMANIA database (http://genemania.org/). Employing the merged PsychENCODE and GTEx gene expression matrix for brain cells, which encompasses a broad spectrum of cell types, including excitatory and inhibitory neurons, major non‐neuronal types (e.g., astrocytes, oligodendrocytes, and microglia), and other developmentally relevant cell types, we sought to identify the enrichment of target gene expression in specific cell types. This was achieved by analyzing differences in gene expression across cell types using the Wilcoxon rank‐sum test, with statistical significance defined as an FDR‐corrected *p* value less than 0.05 and a log_2_(fold‐change) greater than 1.5.

### 2.7 Expression Verification

Immunofluorescence staining was used to determine whether certain encoded proteins of identified genes are enriched in specific brain cell types (using astrocytes as an example). Multiplex immunofluorescence staining was performed at Servicebio (Wuhan, China) according to established protocols. The primary antibodies used for immunofluorescence were against GFAP (Proteintech, 16825‐1‐AP), DNA2 (Proteintech, 18727‐1‐AP), EGFR (Proteintech, 18986‐1‐AP), and VAV3 (Proteintech, 30291‐1‐AP). The cell nucleus was stained with 4′,6‐diamidino‐2‐phenylindole (DAPI). The slides were imaged using a scanner, and the acquired image data were analyzed using CaseViewer software. Glioma samples were obtained at the Neurosurgery Department of the Second Affiliated Hospital of Nanchang University. The excision and preservation of the samples adhered to laboratory standards and strictly followed the Declaration of Helsinki guidelines (as revised in Brazil 2013). All patients who participated in this study signed informed consent forms, and the study was approved by the Ethics Committee of the Second Affiliated Hospital of Nanchang University.

The Human Protein Atlas (HPA, https://www.proteinatlas.org/) database was used to display the protein's subcellular expression and distribution images of immunofluorescence in glioma cell lines (U‐251MG). Furthermore, representative immunohistochemistry (IHC) staining results for proteins in the cerebral cortex were also obtained. Each image displays the staining scores, intensity scores, and fraction of cells with expression of corresponding proteins.

### 2.8 Druggability Evaluation of the Identified Genes

An extensive druggability assessment was conducted for genes identified as potential therapeutic targets, and their druggability was prioritized. For this purpose, the DGIdb database (https://www.dgidb.org/), the DrugBank database (https://go.drugbank.com/), and the Open Targets Platform (https://www.opentargets.org/) were searched to obtain information on drug‐gene interactions and clinical development activities.

Furthermore, in light of the significant causal relationship between the expression of these brain cell type‐specific genes and the risk of glioma, we also conducted a Connectivity Map (CMap) analysis based on the CMap database (https://clue.io) to assess the connectivity between drug compounds and gene expression signatures, aiming to predict the primary compounds capable of modulating gene expression activity [28]. Specifically, we utilized the gene expression signature data from the CMap database, which were generated by treating various types of cells with perturbations, including a diverse array of small molecule compounds, gene overexpression, and gene knockdown reagents, to screen for small molecule compounds that elicited gene expression signatures that were either highly similar or contrasting to those identified in the present study. We also collected the IC50 values of drug compounds and gene expression data from the Genomics of Drug Sensitivity in Cancer (GDSC) and Genomics of Therapeutics Response Portal (CTRP) databases to perform drug sensitivity analysis.

## 3 Results

### 3.1 Two‐Sample MR Analysis Identified Genes Affecting Glioma Risk at the Brain Cell Level

**FIGURE 2:**
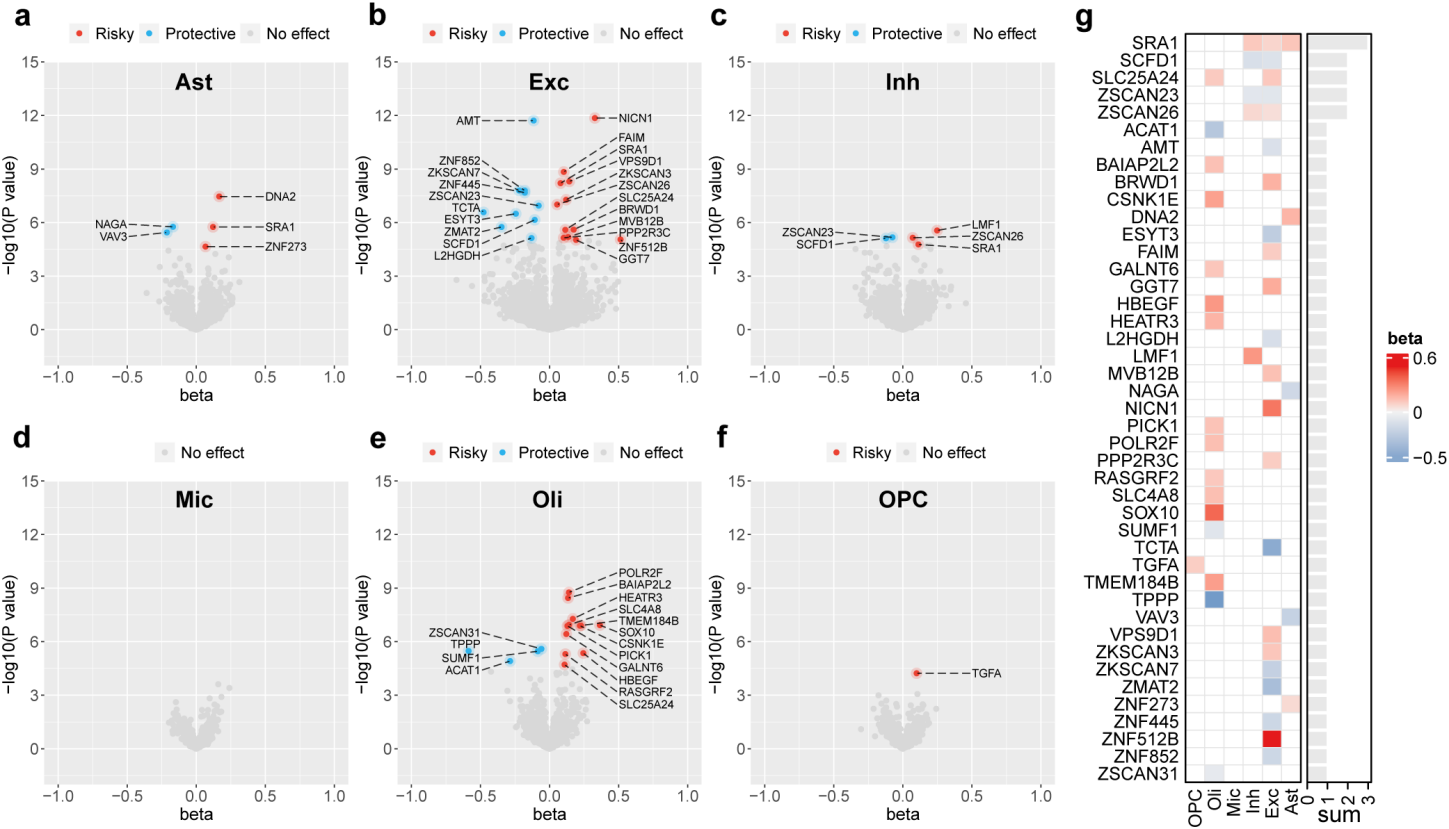
Results of MR analysis for causal effects of genes on all glioma at the brain cell level. (a–f) Volcano plots showing the MR effects of (a) astrocytes, (b) excitatory neurons, (c) inhibitory neurons, (d) microglia, (e) oligodendrocytes, and (f) oligodendrocyte progenitor cells on all glioma. (g) Heat map summarizing the significant MR results in six brain cell types.

After Bonferroni correction, our MR analysis of brain cell type‐specific eQTLs and all glioma subtypes identified a total of 43 gene–cell–outcome associations, specifically 5 genes associated with glioma risk in astrocytes, 22 genes in excitatory neurons, 5 genes in inhibitory neurons, no genes in microglia, 16 genes in oligodendrocytes, and one gene in OPCs (Figure 2a–f). Summarizing the MR results presented in the heatmap, we observed that SRA1, SCDF1, SLC25A24, ZSCAN23, and ZSCAN26 were notably associated with glioma risk across at least two cell types, with consistent MR estimates, indicating potential cross‐cell effects of these genes (Figure 2g).

**FIGURE 3:**
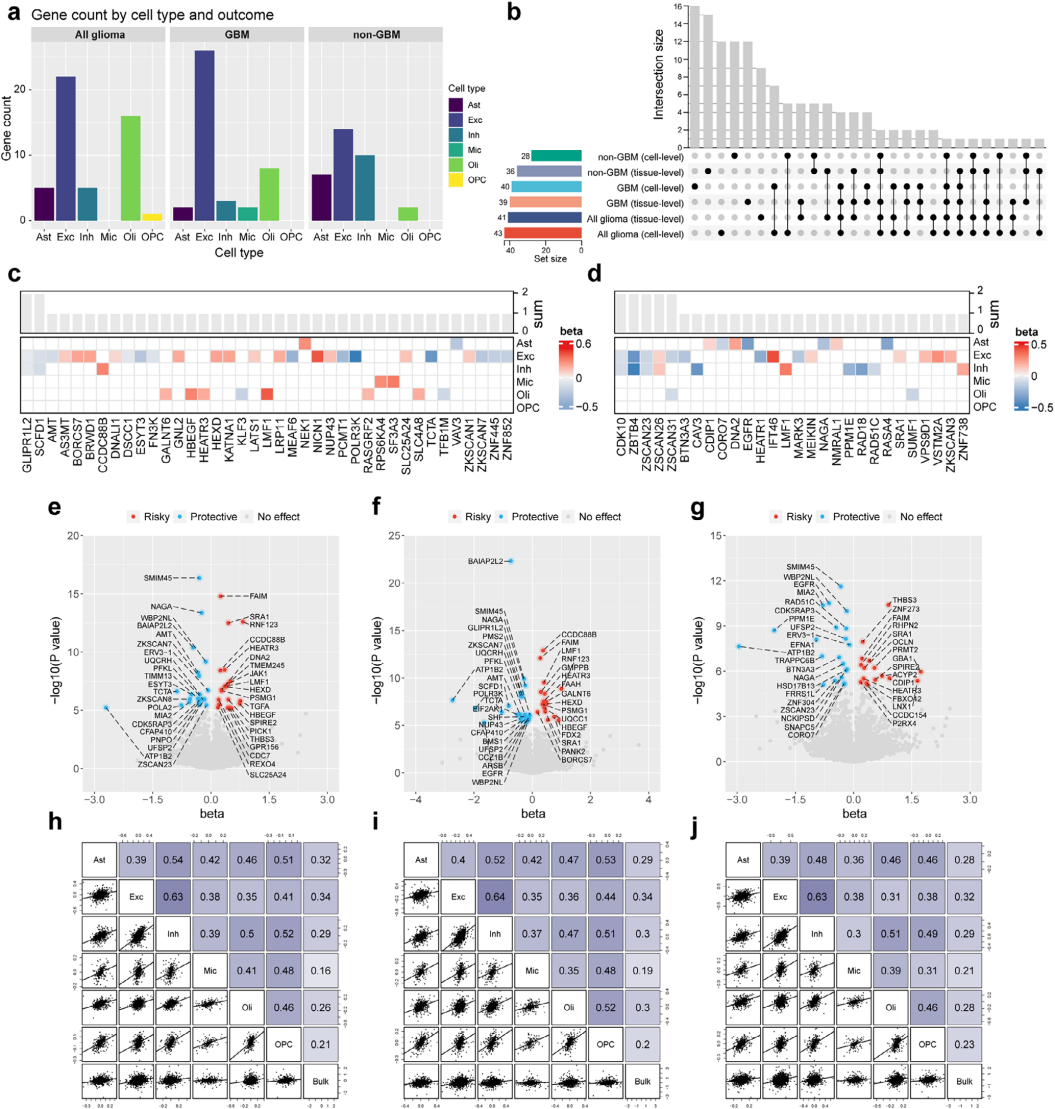
Results of MR analysis for causal effects of genes on glioma subtypes at the brain cell and tissue levels. (a) Number of genes significantly associated genes in each cell type across all glioma, GBM, and non‐GBM subtypes. (b) UpSet plot showing causal genes overlapping among various glioma subtypes or across different levels of eQTL exposure. (c, d) Heatmap summarizing the causal effects of genes on (c) GBM and (d) non‐GBM outcomes. (e–g) Volcano plots showing the brain tissue‐level MR effects of genes on (e) all glioma, (f) GBM, and (g) non‐GBM outcomes. (h–j) Spearman correlation estimates between MR effect sizes of genes on (h) all glioma, (i) GBM, and (j) non‐GBM outcomes.

We also conducted an MR analysis of glioma subtypes. Figure 3a shows the number of significantly associated genes in each cell type across all glioma, GBM, and non‐GBM subtypes. To compare the similarities between brain cell‐specific eQTL MR estimates and those from bulk brain tissue and to identify brain cell‐specific causal genes, we conducted MR analysis on glioma risk, focusing on the same gene expressed in brain tissue (Table S3). An UpSet plot was utilized to quantify the overlap of causal genes among various glioma subtypes or across different levels of eQTL exposure, as depicted in Figure 3b. Upon MR analysis of the brain cell level of eQTL exposure, 17 causal genes identified across all glioma subtypes were found to overlap with GBM and 10 with non‐GBM. Only the LMF1 gene was significantly associated with all three outcomes, whereas a single overlapping causal gene was observed between GBM and non‐GBM. Furthermore, 22 novel causal genes were identified specifically in GBM and 18 in non‐GBM, when compared to all glioma subtypes, indicative of the subtype‐specific nature of these genes. Through comparative analyses of MR at different levels of eQTL exposure, we identified a total of 52 (52/83) genes that exhibited significant causal associations exclusively in cell‐specific eQTL MR analysis, while 31 (31/83) genes were similarly identified in bulk tissue eQTL MR analysis. Heatmaps presenting cell‐specific eQTL MR estimates, focusing on diverse brain cell types, unveiled that GLIPR1L2 and SCDF1 were significantly associated with GBM risk in excitatory and inhibitory neurons (Figure 3c,d). CDK10, ZBTB4, ZSCAN23, and ZSCAN26 were associated with non‐GBM risk in both neuronal types, whereas ZSCAN31 was related to non‐GBM risk, specifically in excitatory neurons and oligodendrocytes. Furthermore, bulk tissue eQTL MR analysis revealed 41, 39, and 36 associations in all glioma, GBM, and non‐GBM cases, respectively, after applying Bonferroni correction (Figure 3e–g).

In subsequent sensitivity analyses, the 13 MR associations exhibited inconsistency in the direction of causality estimates across the three employed MR methods (Table S4). A pleiotropic directionality test, utilizing the MR‐Egger intercept, revealed horizontal pleiotropy (*p* < 0.05) specifically for the ZSCAN26‐ excitatory neurons‐all glioma association (Table S5). Notably, all *p* values > 0.05 for tests of Rucker's *Q* and Cochran's *Q*, suggesting that our findings were unlikely to be influenced by heterogeneity (Table S6). Overall, our MR analyses identified 123 significant associations, whereas 13 nonrobust associations were subsequently excluded, providing compelling evidence that gene expression at the brain cell level is strongly associated with the risk of different glioma subtypes.

Finally, we compared the effect estimates from MR analyses on glioma risk, focusing on the same genes expressed within brain tissue and the disparities observed between distinct cell types. Spearman correlation analyses revealed weak correlations between microglia eQTL and bulk tissue eQTL MR outcomes, whereas the remaining associations demonstrated stronger correlations. Notably, this trend was accentuated with more stringent *p* value threshold adjustments (no threshold, *p* < 0.5, *p* < 0.25), as evident in Figure 3h–j and Figure S1.

### 3.2 Colocalization and Replication Analyses

**FIGURE 4:**
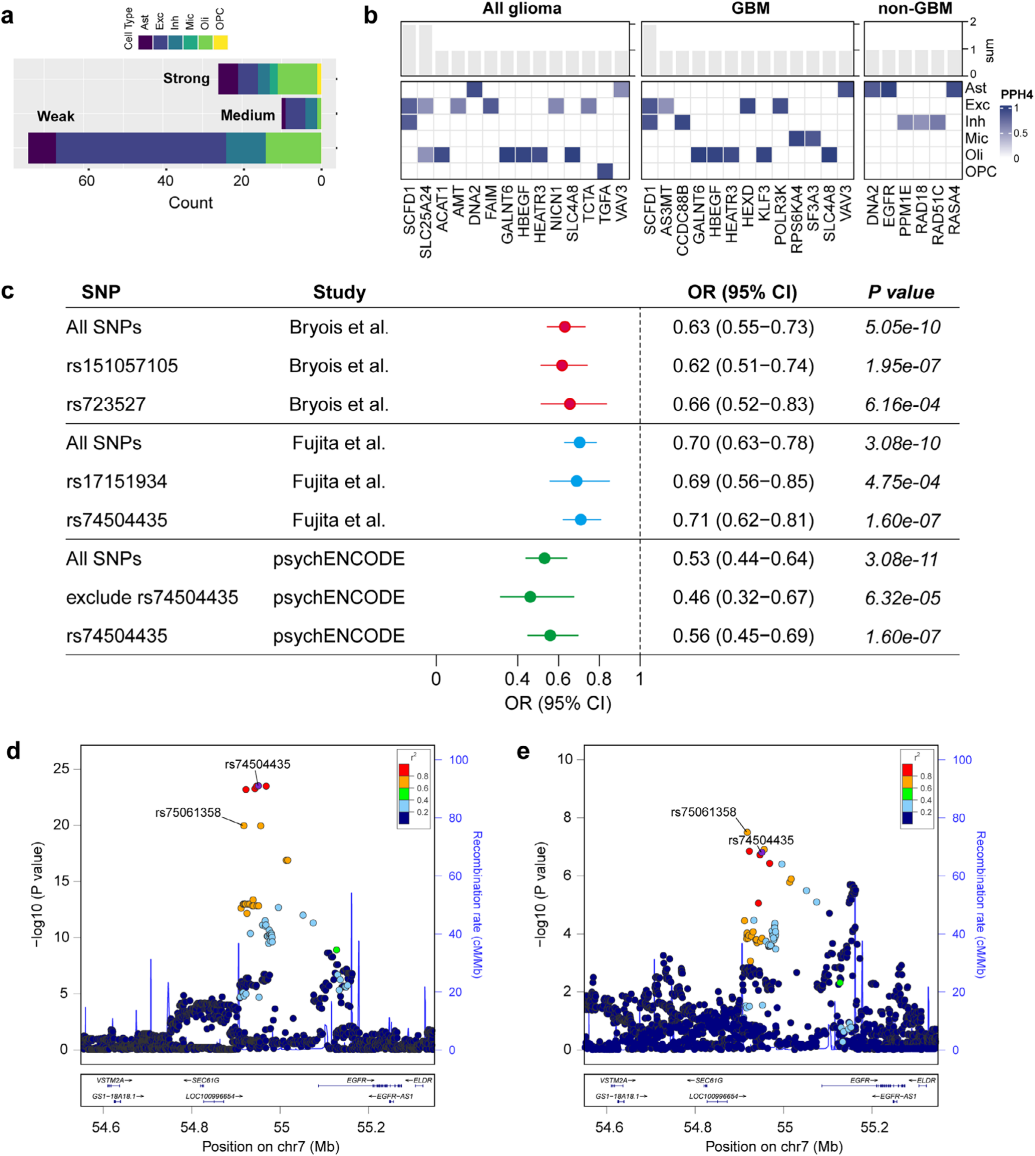
Colocalization results of brain cell‐specific eQTL and glioma GWAS signals at the gene locus. (a) Number of colocalized genes for different evidence. (b) Heatmap summarizing the colocalization results. (c) Results of MR analysis of IVs that served as proxies for EGFR gene expression in astrocytes and brain tissue. (d, e) LocusZoom plots illustrating the (d) astrocyte eQTL and (e) non‐GBM GWAS associations within 200 kb loci flanking rs74504435.

False‐positive results may arise when exposure and outcome are influenced by distinct causal gene variants [29]. Colocalization analysis enabled a more robust and exhaustive interpretation of the relationship between causal genes and disease outcomes, thereby fostering the advancement of targeted drug development. Figure 4a shows that of the 110 MR‐supported associations, 26 exhibited strong colocalization evidence (PPH4 ≥ 80%), 10 exhibited medium colocalization evidence (PPH4 ≥ 50%), and 74 lacked significant colocalization evidence (PPH4 < 50%). Furthermore, replication analyses were conducted for associations with evidence of colocalization, employing data sourced from diverse brain regions, as reported by Bryois et al. Notably, despite a considerable fraction of genes in this study did not have qualifying IVs for MR analysis, the study nonetheless allowed us to validate and corroborate numerous findings to the fullest extent feasible.

Specifically, strong evidence of colocalization for SCDF1 was observed across multiple cell types (including excitatory and inhibitory neurons) and diverse outcomes (all glioma and GBM) (Figure 4b and Table S7). Twenty‐two associations supported by MR and colocalization analyses (involving 16 genes with eligible IVs) were subjected to MR analysis in the replicated dataset, of which 16 associations remained significant (72.73%), partially attesting to the robustness of our results (Table S8).

Furthermore, the comparison of genes with previously identified GWAS associations and MR studies uncovered that EGFR resides within a known glioma risk locus and has been implicated in glioma risk through a multi‐tissue MR study, with the possibility that gene expression in specific tissues differentially influences risk [17]. Our findings indicate that the association between EGFR and non‐GBM risk is cell‐specific and that a substantial portion of the causal effect manifested in tissues can be attributed to astrocytes. To delve into the underlying drivers, we individually conducted an MR analysis of IVs that served as proxies for EGFR gene expression in astrocytes and contrasted them with those for EGFR expression in bulk brain tissues. The analysis revealed rs74504435 as a SNP shared by astrocytes and brain tissues, exhibiting a consistent direction of effect and attaining a minimal *p* value (Figure 4c). This SNP partially mediates the association between EGFR and non‐GBM risk. In replication cohort analyses, EGFR gene expression was proxied by varying IVs, yet the causal effects remained equally significant. Figure 4d depicts the astrocyte eQTL and non‐GBM GWAS associations within 200 kb loci flanking rs74504435.

### 3.3 Identification of Likely Causal Glioma Risk Factors

**FIGURE 5:**
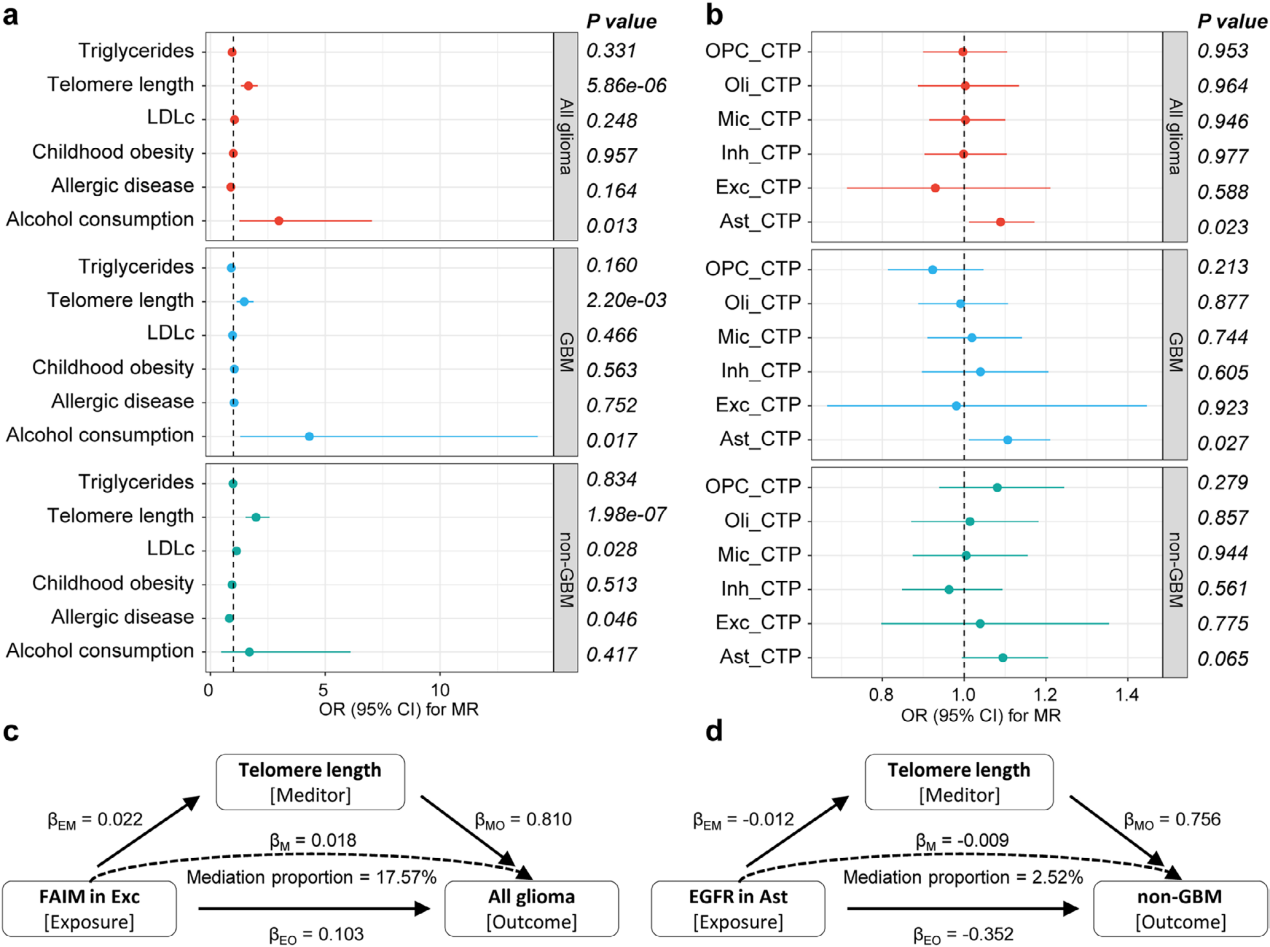
Results of mediation analysis. (a) Results of MR analysis for causal effects of glioma risk factors on glioma outcomes. (b) Results of MR analysis for causal effects of brain cell‐type proportions on glioma outcomes. (c, d) Mediation effect of FAIM in excitatory neurons and EGFR in astrocytes on glioma via risk factors. β_EM_, effects of exposure on mediator; β_EO_, effects of exposure on outcome; β_MO_, effects of mediator on outcome.

To elucidate the potential causal pathways linking causal genes at the brain cell level to glioma outcomes, we initially identified several likely causal glioma risk factors utilizing MR analysis (Figure 5a and Table S9). MR estimates with *p* values less than the Bonferroni‐corrected threshold (0.05/6) were considered statistically significant. Among the six risk factors considered, elongated telomere length exhibited significant associations with the risks of all glioma, GBM, and non‐GBM (OR (95% CI) = 1.66 (1.33 to 2.06), 1.47 (1.15 to 1.88) and 1.99 (1.53 to 2.57), respectively), and the association remained significant at the Bonferroni‐corrected threshold (*p* = 5.86 × 10^−6^, 0.0022 and 1.98 × 10^−7^, respectively). Furthermore, increased alcohol consumption was suggestively associated with an increased risk of glioma and GBM, and decreased LDLc levels and genetically determined increased allergic disease risk were suggestively associated with a decreased risk of non‐GBM (0.05/6 ≤ *p* < 0.05). No significant associations were observed between childhood obesity or triglyceride levels and glioma outcomes (*p* > 0.05). Furthermore, the associations between six brain CTPs and glioma risk were examined. The results tentatively indicated a causal association between elevated astrocyte levels and an increased risk of all glioma and GBM (Figure 5b). In sensitivity analyses, these associations were consistently observed in the same direction of causal estimation across all three methods. The MR‐Egger intercept test specifically indicated the presence of horizontal pleiotropy in the causal association between telomere length and GBM (*p* = 0.043; Table S10). Notably, Cochran's *Q* and Rucker's *Q* tests showed some heterogeneity in IVs of telomere length, allergic disease, childhood obesity, and triglycerides (Table S11). The weighted median approach still supports a causal association between telomere length and glioma risk. Ultimately, we identified telomere length as the causal risk factor for glioma and non‐GBM for subsequent analyses.

### 3.4 Mediation Effect of the Identified Genes on Glioma via Risk Factors

We explored causal associations between 26 genes identified by MR and Bayesian colocalization analyses and telomere length and revealed that a total of four genes—DNA2 and EGFR in astrocytes, FAIM in excitatory neurons, and HEATR3 in oligodendrocytes—were associated with telomere length (Table 1 and Figure 5c,d). The investigation further demonstrated that these four genes exhibited distinct mediating effects through telomere length. Specifically, the mediation effect of EGFR was −0.009 (95% CI: −0.018 to 9.44 × 10^−5^), accounting for a relatively weak proportion (2.52%) of the total effect. In contrast, the mediation effect of FAIM was 0.018 (95% CI: 0.01 to 0.026), contributing 17.57% of the total effect. Additionally, it was observed that the mediation effects of DNA2 and HEATR3 via telomere length were in the opposite direction to their respective total effects, suggesting a potential masking effect, wherein these genes may diminish part of the glioma risk effect size by reducing telomere length.

**TABLE 1:** Results of mediation analysis for genes on glioma outcomes via risk factors.

Mediator	Cell type	Exposure	Outcome	Total effect	Direct effect EM	Direct effect MO	Mediation effect	Mediation proportion (%) (95% CI)
				Beta (95% CI)	Beta (95% CI)	Beta (95% CI)	Beta (95% CI)	
Telomere length	Ast	DNA2	All glioma	0.166 (0.107 to 0.225)	−0.011 (−0.017 to −0.004)	0.810 (0.518 to 1.101)	−0.009 (−0.015 to −0.002)	—
	Exc	FAIM	All glioma	0.103 (0.070 to 0.136)	0.022 (0.016 to 0.029)	0.810 (0.518 to 1.102)	0.018 (0.01 to 0.026)	17.57%
	Oli	HEATR3	All glioma	0.169 (0.108 to 0.229)	−0.018 (−0.026 to −0.010)	0.810 (0.518 to 1.103)	−0.015 (−0.023 to −0.006)	—
	Oli	HEATR3	GBM	0.194 (0.120 to 0.269)	−0.018 (−0.026 to −0.010)	0.580 (0.227 to 0.933)	−0.011 (−0.019 to −0.003)	—
	Ast	DNA2	Non‐GBM	0.218 (0.141 to 0.295)	−0.011 (−0.017 to −0.004)	0.756 (0.322 to 1.190)	−0.008 (−0.015 to −0.001)	—
	Ast	EGFR	Non‐GBM	−0.352 (−0.461 to −0.242)	−0.012 (−0.021 to −0.002)	0.756 (0.322 to 1.191)	−0.009 (−0.018 to 9.44e‐5)	2.52%

*Note:* Total effect is the effect of exposure on outcome derived from two‐sample MR. Direct effect EM is the effect of exposure on mediator. Direct effect EO is the effect of mediator on outcome.

### 3.5 Genetic Mutation, Gene Enrichment, PPI Network, and Expression Analyses

**FIGURE 6:**
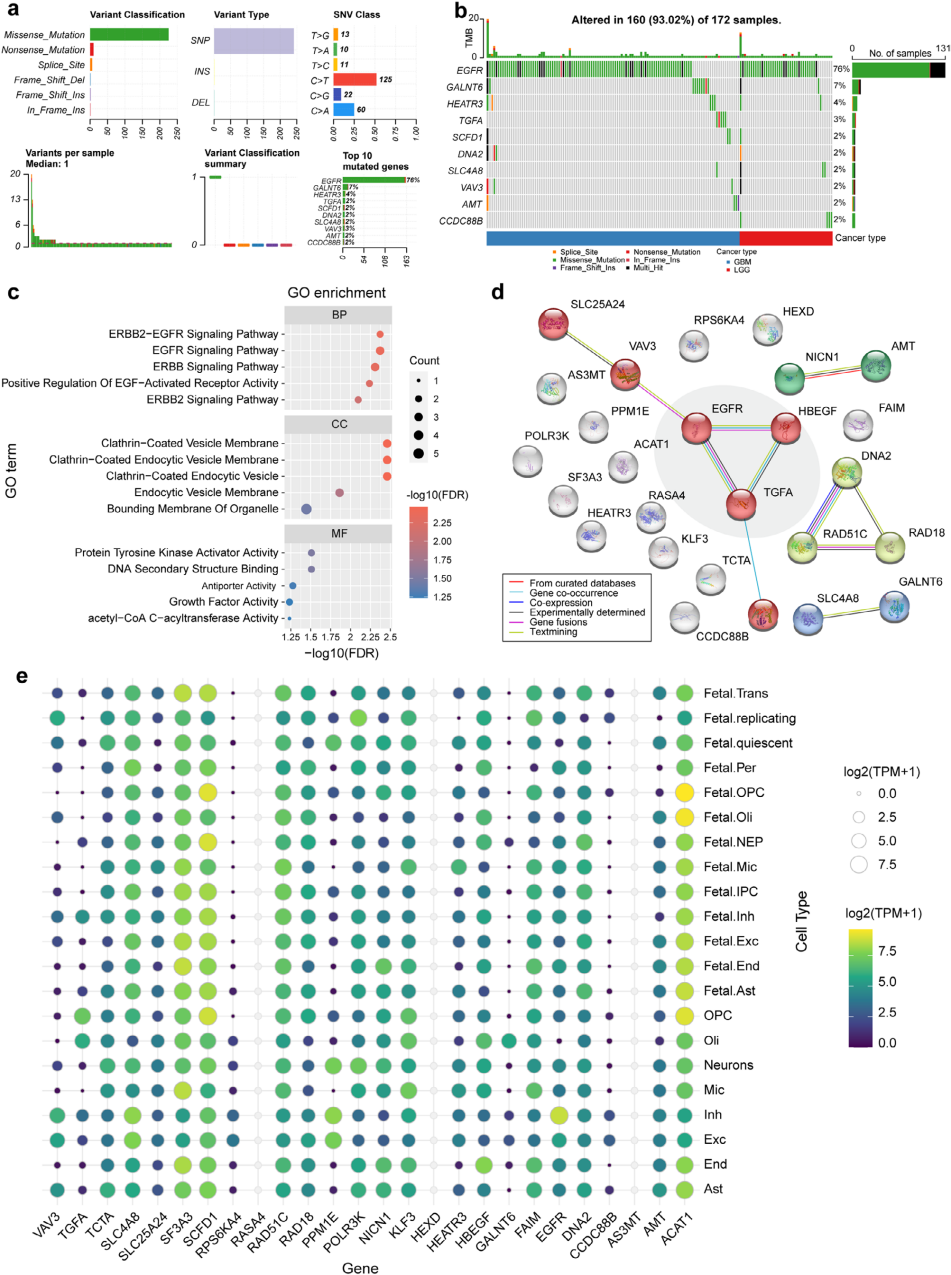
Results of bioinformatic analyses. (a) Counts and types of single nucleotide variants in the glioma sample and gene levels. (b) Waterfall plot showing the mutation distribution of the top 10 mutated genes in the sample set of gliomas. (c) Results of GO enrichment analysis of the identified genes. (d) PPI network of the identified genes. (e) Gene expression in different brain cell types.

Figure 6a summarizes the count and type of single nucleotide variant (SNV) in the glioma sample and gene levels. A missense mutation was the most common variant classification, and most of the variant types were C > T SNP or C > A SNP. However, the number of median variants per sample is low. Figure 6b shows the count and percentage of variants in the top 10 mutated genes. EGFR has the highest mutation frequency (76%), followed by GALNT6 (7%), while other genes have a lower mutation frequency (< 5%). Most causal genes underwent copy number variation (CNV) in glioma samples, with AS3MT, DNA2, and SCDF1 being predominantly heterozygous deletion, while RASA4 and EGFR were predominantly heterozygous and homozygous amplification. Most causal genes underwent CNV in glioma samples, with AS3MT, DNA2, and SCDF1 being predominantly heterozygous deletion, while RASA4 and EGFR were predominantly heterozygous and homozygous amplification. In addition, genes such as SFA3, VAV3, SLC25A24, and ACTA1 have different predominant CNV classes in GBM and LGG (Figure S2).

In terms of BP, most of the genes related to the epidermal growth factor (EGF) receptor family of receptor tyrosine kinases were enriched predominantly in specific pathways. In terms of CC, these genes are predominantly associated with the clathrin‐coated (endocytic) vesicle membrane and the bounding membrane of organelles. In terms of MF, these genes exhibited genetically enriched protein tyrosine kinase activator activity and DNA secondary structure binding function, among others (Figure 6c). KEGG enrichment analysis revealed that these genes are implicated in the ErbB signaling pathway, glyoxylate and dicarboxylate metabolism, estrogen signaling pathway, Ras signaling pathway, and glioma (Figure S3a). Furthermore, GWAS Catalog library enrichment analysis indicated that these genes were associated with the physiopathology of glioblastoma (glioma), age at menopause, autoimmune traits, and white matter microstructure (fractional anisotropy) (Figure S3b).

Furthermore, the identified genes were input into the STRING database to construct a PPI network. Figure 6d shows the interactions in the 26‐node 11‐sided PPI network (interaction score > 0.4). When integrated with the findings from gene enrichment analysis, the interactions among EGFR, HBEGF, and TGFA emerged as the central elements of the PPI network. They were involved in the preponderance of enriched entries. The PPI network constructed from the GeneMANIA database showed similar results (Figure S3c). Twenty‐three protein‐coding genes were subjected to expression analysis, of which RASA4, HEXD, and AS3MT expression were not detected. Notable cell‐specific gene expression patterns were observed, with ACAT1 being enriched in developing oligodendrocyte cell lines, CCDC88B in excitatory neurons, EGFR in inhibitory neurons, HBEGF and GALNT6 in oligodendrocytes, NICN1 in astrocyte cells, KLF3 in microglia, and TGFA exhibiting significant enrichment in OPCs, among others (Figure 6e and Table S12). We also examined sex‐based differences in gene expression across various types of gliomas and brain cell types. The results indicated that AMT, AS3MT, EGFR, NICN1, and POLR3K exhibited significant sex differences in expression within glioma tissues. Additionally, SF3A3, EGFR, AS3MT, SCFD1, POLR3K, and SCL4A8 displayed sex‐specific expression patterns in conjunction with certain brain cell types (Figure S4). Multiplex immunofluorescence staining revealed relatively low expression levels of DNA2, EGFR, and VAV3, which complicated the determination of their specific enrichment within astrocytes (Figure S5). Representative IHC staining results for proteins in the cerebral cortex displayed staining scores, intensity scores, and fraction of cells (including neuronal cells and glial cells) with expression of corresponding proteins (Figure S6). Images of immunofluorescence in U‐251MG show the subcellular localization of several proteins (Figure S7).

### 3.6 Druggability Evaluation

We evaluated the identified genes for their druggability potential and prioritized them according to the strength of colocalization evidence, the statistical significance of replication analysis, and the existence of drug‐gene interactions or ongoing drug development activities. Among the 26 genes associated with glioma risk, 10—ACAT1, AMT, AS3MT, DNA2, EGFR, HBEGF, RPS6KA4, SF3A3, SLC4A8, and TGFA—were recognized as potential targets for drug development or exhibited drug‐gene interactions (Table 2 and Table S13), thereby classifying them as tier 1 drug targets in our study. Therapeutics directed against EGFR have been extensively employed in glioma therapy. Clinical trials have assessed agents related to other genes for treating various diseases, yet their application in glioma therapy remains unexplored. For instance, targeting HBEGF is utilized in treating peritoneal neoplasm, ovarian neoplasm, and fallopian tube carcinoma. FEPIXNEBART, a drug that targets TGFA, is administered for the management of osteoarthritis, low back pain, and neuropathic pain, among other conditions. Genes lacking documented drug‐gene interactions or drug development activities were assigned to tier 2 if strong colocalization evidence and statistically significant replication analyses were present; otherwise, they were classified as tier 3 drug targets.

**TABLE 2:** Druggability evaluation of the potential brain cell type‐specific therapeutic targets.

Gene	Description	Gene categories^a^	Brain cell type	Diseases	Colocalization evidence^b^	Replication analysis^c^	Drug‐gene interaction	Drug development activities		Druggability priority
							DGIdb	DrugBank	Open Targets	
ACAT1	Acetyl‐CoA Acetyltransferase 1	Enzyme	Oli	Glioma	Strong	—	Yes	Yes	—	Tier1
AMT	Aminomethyltransferase	Enzyme	Exc	Glioma	Medium	—	Yes	Yes	—	Tier1
AS3MT	Arsenite Methyltransferase	—	Exc	GBM	Medium	—	—	Yes	—	Tier1
CCDC88B	Coiled‐Coil Domain Containing 88B	—	Inh	GBM	Strong	Yes	—	—	—	Tier2
DNA2	DNA Replication Helicase/Nuclease 2	Enzyme	Ast	Glioma/non‐GBM	Strong	Yes	Yes	—	—	Tier1
EGFR	Epidermal Growth Factor Receptor	Clinically actionable, Drug resistance, Druggable genome, Kinase, Tyrosine kinase, Cell surface	Ast	Non‐GBM	Strong	Yes	Yes	Yes	Yes	Tier1
FAIM	Fas Apoptotic Inhibitory Molecule	—	Exc	Glioma	Strong	Yes	—	—	—	Tier2
GALNT6	Polypeptide N‐Acetylgalactosaminyltransferase 6	Enzyme	Oli	Glioma/GBM	Strong	Yes	—	—	—	Tier2
HBEGF	Heparin Binding EGF Like Growth Factor	Druggable genome, Cell surface, Growth factor	Oli	Glioma/GBM	Strong	No	Yes	—	Yes	Tier1
HEATR3	HEAT Repeat Containing 3	—	Oli	Glioma/GBM	Strong	No	—	—	—	Tier3
HEXD	Hexosaminidase D	—	Exc	GBM	Strong	Yes	—	—	—	Tier2
KLF3	KLF Transcription Factor 3	—	Oli	GBM	Strong	Yes	—	—	—	Tier2
NICN1	Nicolin 1, Tubulin Polyglutamylase Complex Subunit	—	Exc	Glioma	Medium	—	—	—	—	Tier3
POLR3K	RNA Polymerase III Subunit K	—	Exc	GBM	Strong	—	—	—	—	Tier3
PPM1E	Protein Phosphatase, Mg2+/Mn2+ Dependent 1E	Kinase	Inh	Non‐GBM	Medium	Yes	—	—	—	Tier3
RAD18	RAD18 E3 Ubiquitin Protein Ligase	Enzyme, DNA repair	Inh	Non‐GBM	Medium	No	—	—	—	Tier3
RAD51C	RAD51 Paralog C	Clinically actionable, Kinase, DNA repair	Inh	Non‐GBM	Medium	—	—	—	—	Tier3
RASA4	RAS P21 Protein Activator 4	Kinase	Ast	Non‐GBM	Strong	—	—	—	—	Tier3
RPS6KA4	Ribosomal Protein S6 Kinase A4	—	Mic	GBM	Strong	Yes	Yes	Yes	—	Tier1
SCFD1	Sec1 Family Domain Containing 1	Druggable genome, Transcription factor, Kinase, Serine threonine kinase, Clinically actionable	Exc/Inh	Glioma/GBM	Strong	Yes	—	—	—	Tier2
SF3A3	Splicing Factor 3a Subunit 3	—	Mic	GBM	Strong	—	—	Yes	—	Tier1
SLC25A24	Solute Carrier Family 25 Member 24	—	Exc/Oli	Glioma	Medium	Yes	—	—	—	Tier3
SLC4A8	Solute Carrier Family 4 Member 8	External side of plasma membrane	Oli	Glioma/GBM	Strong	No	—	Yes	—	Tier1
TCTA	T Cell Leukemia Translocation Altered	—	Exc	Glioma	Medium	—	—	—	—	Tier3
TGFA	Transforming Growth Factor Alpha	Druggable genome, Cell surface, Growth factor	OPC	Glioma	Strong	Yes	Yes	—	Yes	Tier1
VAV3	Vav Guanine Nucleotide Exchange Factor 3	Kinase	Ast	Glioma/GBM	Strong	—	—	—	—	Tier3

^a^ Gene categories in DGIdb refer to a set of genes belonging to a group that is deemed to be potentially druggable.

^b^ Colocalization evidence was considered strong if at least one gene–cell–outcome association was present.

^c^ Replication analysis was considered positive if at least one gene–cell–outcome association was significant in the replication dataset.

From the CMap database, we retrieved the expression signatures (encompassing gene overexpression and knockdown) for 13 genes and screened for compounds that exhibited a high degree of similarity or opposition to these signatures, with enrichment scores exceeding 95 (indicating similarity) or falling below −95 (indicating opposition) (Table S14). Drug sensitivity analysis revealed that increased expression levels of genes such as TGFA, SLC25A24, EGFR, and HBEGF are associated with resistance to most chemotherapy drugs. Conversely, elevated expression of genes, including ACAT1, DNA2, and NICN1, indicates increased sensitivity to drugs (Figure S8).

## 4 Discussion

Through this investigation, leveraging eQTL data derived from distinct brain cell types and employing analyses including MR and colocalization analyses, we, for the first time, elucidate the causal impact of gene expression within specific brain cell types on glioma risk and its various subtypes. In contrast to tissue‐level findings, our study uncovered novel causal linkages that underscore the cell‐specific regulatory functions in gliomagenesis. This observation aligns with the recent spotlight on cellular interactions within the tumor microenvironment and cancer neuroscience, proposing that brain cells are not solely passive entities influenced by gliomas but may actively contribute to regulating gliomagenesis. Ultimately, our study identified 26 potential therapeutic targets specific to distinct brain cell types in glioma, thereby offering novel insights into targeted drug development strategies.

In previous research, a widely adopted strategy for identifying disease risk genes has been the integration of tissue‐level eQTL datasets with GWAS summary statistics. One transcriptome‐wide association study utilized a brain tissue eQTL dataset from the Genotype‐Tissue Expression (GTEx) project [30]. Additionally, several studies focused on glioma‐related eQTL datasets [31, 32], while another incorporated blood eQTL dataset [11]. Although these investigations successfully identified risk genes associated with glioma, they did not fully encapsulate the influence of cell‐specific regulatory mechanisms on causal relationships. Particularly in brain tissue, the intricacy of cell types stands out as a defining characteristic. By employing innovative MR analysis focused on brain cell type‐specific cis‐eQTL datasets, we identified 110 significant and robust gene–cell–outcome associations, encompassing 83 unique genes. Except for EGFR, HEATR1, VAV3, GALNT6, LMF1, and HEATR3, which have been reported in previous studies, the remaining risk genes represent new findings. Furthermore, compared to the findings from brain tissue‐level MR analysis, 52 genes exhibited significant causal effects exclusively at the brain cell level. These findings emphasize the critical importance of identifying glioma risk genes at the cellular level, underscoring the essential role of cell‐specific regulation in gliomagenesis. Compared with conventional tissue‐level analyses, investigations conducted at the cellular level can reveal greater biological complexity.

However, several well‐established risk genes, such as mutated IDH and TERT [1, 33], were not identified in our study. The absence of these genes can be attributed to several factors: (1) Data limitations. As described by Fujita et al., the mapping of cis‐eQTLs underwent a certain screening process [19]. Consequently, genes that were not expressed or were expressed at low levels in brain cells or whose cis‐eQTLs did not meet quality control standards could not be included in our analysis. (2) Methodological factors. It is essential to establish a set of screening criteria to ensure that IVs adhere to the key assumptions of MR studies [25]. Thus, genes lacking qualifying IVs were excluded from further analysis. (3) Research background. While we acknowledge the significant roles of mutated genes such as IDH in glioma development, our emphasis lies on how gene expression changes—resulting from gene amplification or deletion—affect glioma risk. Alterations in gene expression do not always correlate with the presence of mutations in those genes.

A pivotal finding was the observation that most genes demonstrated causal effects exclusively in distinct brain cell types, notably astrocytes, excitatory and inhibitory neurons, and oligodendrocytes. In contrast, microglia and OPCs were associated with fewer causal genes. This phenomenon may stem from substantial disparities in the number of eQTLs among cell types, which the varying proportions of cell types can partially explain. In this study, the primary data was on utilizing single‐nucleus‐derived eQTLs from the neocortex, where neurons constitute the predominant cell type, in contrast to microglia and oligodendrocytes, which are less prevalent [19]. Notably, a limited set of genes exhibited consistent causal effects across diverse cell types, including SRA1, SCFD1, and SLC25A24. These genes have the potential to modulate an array of brain‐related processes and diseases, such as neurite outgrowth and Parkinson's disease. Yet, no extant research has demonstrated their direct involvement in glioma pathogenesis [34, 35, 36].

In addition, MR analysis revealed that the causal effect is specifically distinct among glioma subtypes, notably distinguishing GBM from non‐GBM, given their shared sole causal gene, LMF1. Notably, the risk gene count for all glioma is lower than that of GBM and non‐GBM combined. A plausible explanation for this observation is the subtype‐specific nature of genetic variation characteristics in gliomas. A meta‐analysis of GWAS confirmed 25 risk loci associated with gliomas. Notably, only nine of these risk loci were shared between GBM and non‐GBM subtypes [11], indicating that a significant number of risk loci exhibit subtype‐specific characteristics. Thus, given subtype‐specific genetic variation, the extent to which gene expression is associated with glioma risk will vary across subtypes. This results in significant associations between certain genes and glioma risk, which are observed only in specific subtypes. In contrast, when conducting a comprehensive analysis of all glioma, the overall reduction in risk association levels may result in a lack of statistical significance.

The 26 genes, encompassing 36 associations supported by MR and Bayesian colocalization analyses, were deemed potential therapeutic targets for brain cell types. Among these, we focused specifically on EGFR, a tyrosine kinase receptor that is extensively associated with the occurrence and progression of gliomas [37]. Amplification of EGFR and active mutant EGFRvIII occurs frequently in gliomas [38]. Aberrant EGFR signaling is a major driver of glioma invasion and angiogenesis [39]. Additionally, EGFR signaling regulates the proliferation, differentiation, and maturation of astrocytes [40, 41], which is important for understanding gliomagenesis, as astrocytes can act as cells of origin for gliomas [42]. Aberrant EGFR signaling may lead astrocytes to generate pro‐tumor effects or even tumor entities. Two eQTL MR studies conducted at the brain cell level revealed that elevated EGFR gene expression in astrocytes correlated with a decreased risk of non‐GBM, a finding corroborated by bulk brain tissue MR analysis and a previously published study [17].

Further detailed analysis disclosed that rs74504435 served as a top SNP for the EGFR eQTL in both astrocytes and brain tissues. The 7p11.2 association with non‐GBM that is marked by rs75061358 localizes to loci near EGFR and exhibits LD with rs74504435 [11]. In the replication dataset, the top SNP for astrocyte EGFR eQTL was identified as rs723527, a known glioma risk locus that is also situated at 7p11.2 [43]. These findings offer a novel perspective for investigating the role of EGFR in glioma pathogenesis. A plausible explanation for the variations in the IVs of astrocyte EGFR gene expression between the two cohorts could be their origin from distinct brain regions characterized by varying levels of eQTL activity, potentially influencing the subsequent outcomes of LD removal.

Increased telomere length was identified as a risk factor for glioma, which aligns with previous studies [16, 23, 44, 45]. The influence of certain genes, such as DNA2, EGFR, FAIM, and HEATR3, on glioma risk, which is mediated through alterations in telomere length, enhances the elucidation of pathogenic mechanisms and introduces novel strategies for targeting telomere‐related pathways. DNA2 is a multifunctional enzyme critical to several DNA metabolic pathways, including Okazaki fragment processing (OFP), replication of centromeric DNA, checkpoint activation, telomere homeostasis, and DNA double‐strand break (DSB) repair [46]. Although the precise molecular functions of DNA2 in astrocytes are unclear, and direct evidence linking it to glioma development is lacking, previous studies suggest a dual role for DNA2 in different cancer contexts: it acts as a tumor suppressor by maintaining genome integrity to prevent tumor transformation [47, 48], while also facilitating the survival of cancer cells during DSB repair and replication stress [49, 50]. EGFR‐AMPK signaling has been documented to enhance telomerase activity and sustain telomere length, consequently stimulating the proliferation and immortalization of glioma cells [51]. FAIM is an evolutionarily highly conserved death receptor antagonist that modulates apoptotic cell death and mechanisms of neuronal differentiation [52]. Research has shown that FAIM is implicated in the development of various cancers. For example, FAIM regulates autophagy induction in lung adenocarcinoma cells, thereby promoting cell proliferation [53]. Elevated FAIM expression has been linked to poor survival outcomes in multiple myeloma patients, contributing to the development of multiple myeloma cells [54]. However, the specific role of FAIM in glioma remains largely unexplored. HEATR3, also known as symportin 1 (SYO1), is involved in both ribosomal protein transport and ribosome biogenesis [55]. Single nucleotide polymorphisms (SNPs) at the HEATR3 locus have been reported to be associated with the development of esophageal squamous cell carcinoma [56], testicular germ cell tumor [57], and glioma [11]. A recent study also found that HEATR3 is involved in cell proliferation, metastasis, and cell cycle development in bladder cancer, exerting a pro‐tumourigenic role [58].

Bioinformatic analyses have significantly advanced our comprehension of the pathogenic mechanisms that underlie these potential therapeutic targets. HBEGF and TGFA serve as established ligands for EGFR, and their intricate interplay triggers a cascade of signaling pathways implicated in glioma development [59]. Interestingly, several genes, including AMT, AS3MT, EGFR, NICN1, POLR3K, SCFD1, SF3A3, and SCL4A8, exhibit sex‐specific expression patterns in various types of gliomas or brain cell types. Reports have shown that most glioma histomorphologies are 30%–50% more prevalent in males and that there is a sex difference in the genetic risk of gliomas [60]. Our analyses further elucidate the underlying biological processes underlying this risk. Ultimately, we categorized the druggability of these potential therapeutic targets into three distinct groups, leveraging insights gained from colocalization analysis, replication analysis, and clinical drug development endeavors. This approach has been extensively employed in drug target MR studies [61, 62, 63]. Additionally, CMap analysis, which leverages perturbations in gene expression, is extensively utilized in drug repurposing and target identification, effectively circumventing the constraints of traditional drug discovery about cost, duration, and risk [64, 65].

Although our study was productive, several limitations exist. The data utilized in this study relied solely on publicly accessible eQTL and GWAS datasets, primarily sourced from European populations, potentially constraining the generalizability of our findings. Differences in the pathogenesis of gliomas among various ethnic backgrounds may exist, as may their gene expression profiles, which leads to the possibility that the associations between brain cell type‐specific gene expression and glioma risk may differ accordingly, necessitating validation across broader demographics. To date, cohort sample sizes have been comparatively modest when juxtaposed against eQTL studies involving large brain tissues, constrained by the prohibitive cost of single‐cell sequencing, thereby restricting the statistical ability to uncover cell‐specific eQTLs [66]. Furthermore, numerous eQTLs have been observed to be functional solely within a specific cell subtype or brain region, suggesting that future investigations into cell‐specific eQTLs across diverse brain region cell subtypes may facilitate a deeper exploration of causal genes and potential drug targets [19]. Currently, the histological classification of gliomas has been partially superseded by molecular typing. The WHO CNS5 classification system categorizes adult diffuse gliomas into three distinct types: astrocytoma, IDH‐mutant; oligodendroglioma, IDH‐mutant and 1p/19q‐codeleted; and GBM, IDH‐wildtype [1]. A GWAS by molecular subtype could identify additional germline variants [67]. The glioma GWAS data used in this study fully classified gliomas into GBM and low‐grade non‐GBM, necessitating the inclusion of molecular subtypes in subsequent investigations. However, although this study identified several potential pathogenic genes, the functional validation of these genes in cellular and animal models remains unfulfilled. Future research endeavors must elucidate the precise roles of these genes in glioma development in conjunction with laboratory studies. Hence, caution should be exercised when therapeutic recommendations derived from MR analysis results are considered.

## 5 Conclusions

Based on MR analysis, Bayesian colocalization analysis, mediation analysis, and a series of bioinformatics analyses, our findings reveal associations between genetic regulation of gene expression in specific brain cell types and glioma risk, providing new clues to glioma etiology and enhancing the understanding of how brain cell types, as well as functional activities, influence glioma pathogenesis. We identified potential brain cell type‐specific therapeutic targets for glioma, providing new ideas for developing targeted glioma drugs.

## Author Contributions

K.H.: conceptualization, funding acquisition, methodology, project administration, supervision. X.Z.: conceptualization, funding acquisition, project administration, supervision. J.G.: formal analysis, investigation, methodology, software, visualization, writing – original draft, writing – review and editing. J.C.: data curation, writing – review and editing. K.W.: visualization, writing – review and editing. Y.L.: investigation, writing – review and editing.

## Ethics Statement

All relevant institutional review boards approved the data sources, and no additional ethical approval was required for the use of GWAS summary statistics in this study. Additionally, the excision and preservation of the glioma samples adhered to laboratory standards and strictly followed the Declaration of Helsinki guidelines (as revised in Brazil 2013).

## Consent

All patients who participated in this study signed informed consent forms and the study has been approved by the Ethics Committee of the Second Affiliated Hospital of Nanchang University (IIT‐D‐2022‐003).

## Conflicts of Interest

The authors declare no conflicts of interest.

## Supporting information


Figure S1.

Table S1.

## Data Availability

All data sources used in this study can be acquired from the original studies that are mentioned in the text. Cell type‐level eQTL summary statistics are available at Synapse (https://doi.org/10.7303/syn52335732) and Zenodo (https://doi.org/10.5281/zenodo.5543734). GWAS summary statistics for glioma can be downloaded from the European Genome‐phenome Archive (EGA) (https://ega‐archive.org/). GWAS summary statistics for brain CTPs are available at Zenodo (https://doi.org/10.5281/zenodo.7604233). GWAS summary statistics for glioma risk factors are publicly available through the IEU Open GWAS Project database (https://gwas.mrcieu.ac.uk/). The merged PsychENCODE and GTEx gene expression matrix for brain cells can be downloaded from http://resource.psychencode.org/. The gene mutation data of samples from glioma were acquired from the UCSC Xena website (https://xenabrowser.net/datapages/).
