# Supplementary material for: Identification of Brain Cell Type‐Specific Therapeutic Targets for Glioma From Genetics
Source: CNS Neurosci Ther. 2024 Dec 25;30(12):e70185. doi: 10.1111/cns.70185 (PMC11669572; doi:10.1111/cns.70185)
Supplement: Supplementary file 1 — Figure S1. [file CNS-30-e70185-s002.docx]

***Supplementary Figures***

Supplementary Fig.1 | **Spearman correlation estimates between MR effect sizes of genes on glioma outcomes under different P value screening conditions**. **a-c,** Spearman correlation estimates between MR effect sizes of genes on (**a**) all glioma, (**b**) GBM, and (**c**) non-GBM outcomes under screening conditions with P values <0.5. **d-f,** Spearman correlation estimates between MR effect sizes of genes on (**d**) all glioma, (**e**) GBM, and (**f**) non-GBM outcomes under screening conditions with P values <0.25.


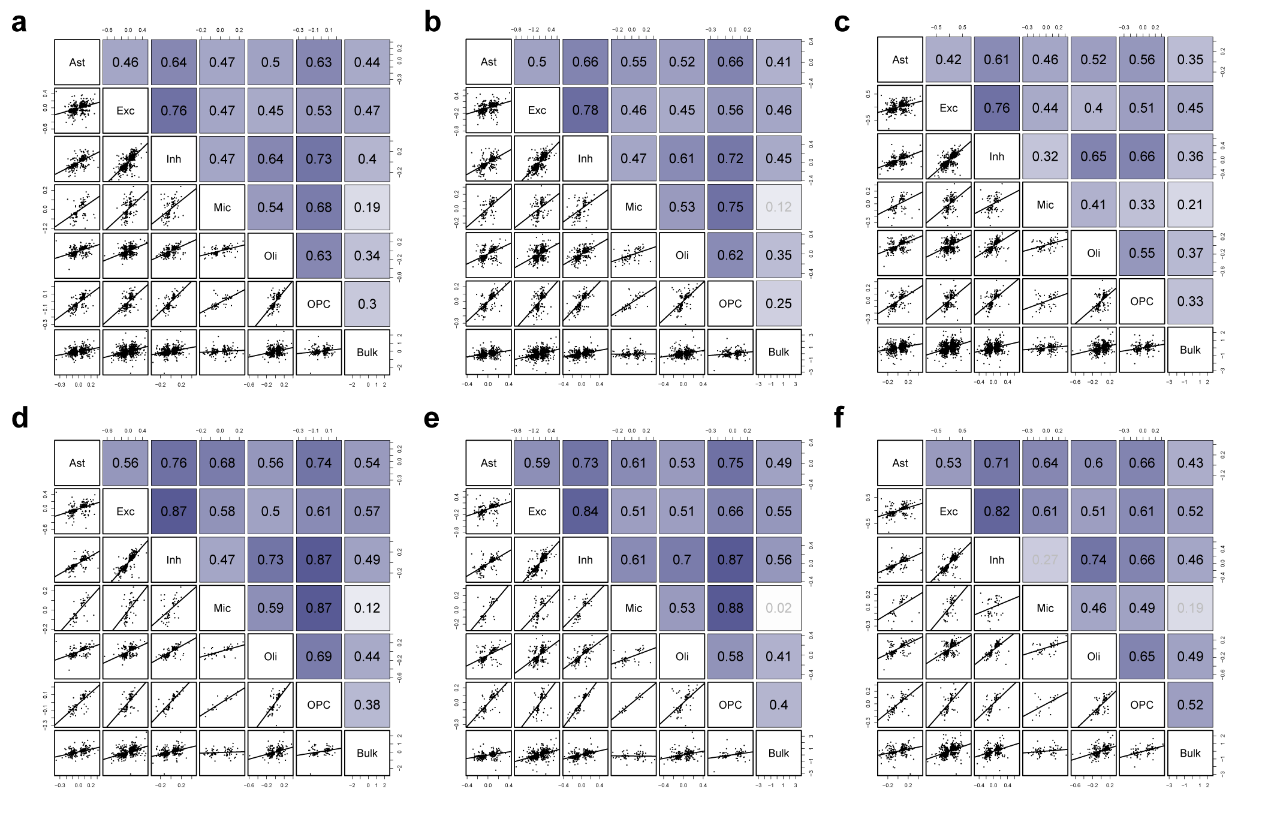


Supplementary Fig.2 | **The summary of genes’ CNV in glioma samples. a,** A global profile showing the constitution of the Heterozygous/Homozygous CNV of each gene in glioma. A pie represents the proportion of different types of CNV of one gene, and different color represents different CNV type. **b,** Bubble plots representing the percentage of homozygous CNV, including homozygous amplification and deletion of each gene. **c,** Bubble plots representing the percentage of heterozygous CNV, including heterozygous amplification and deletion of each gene.

**
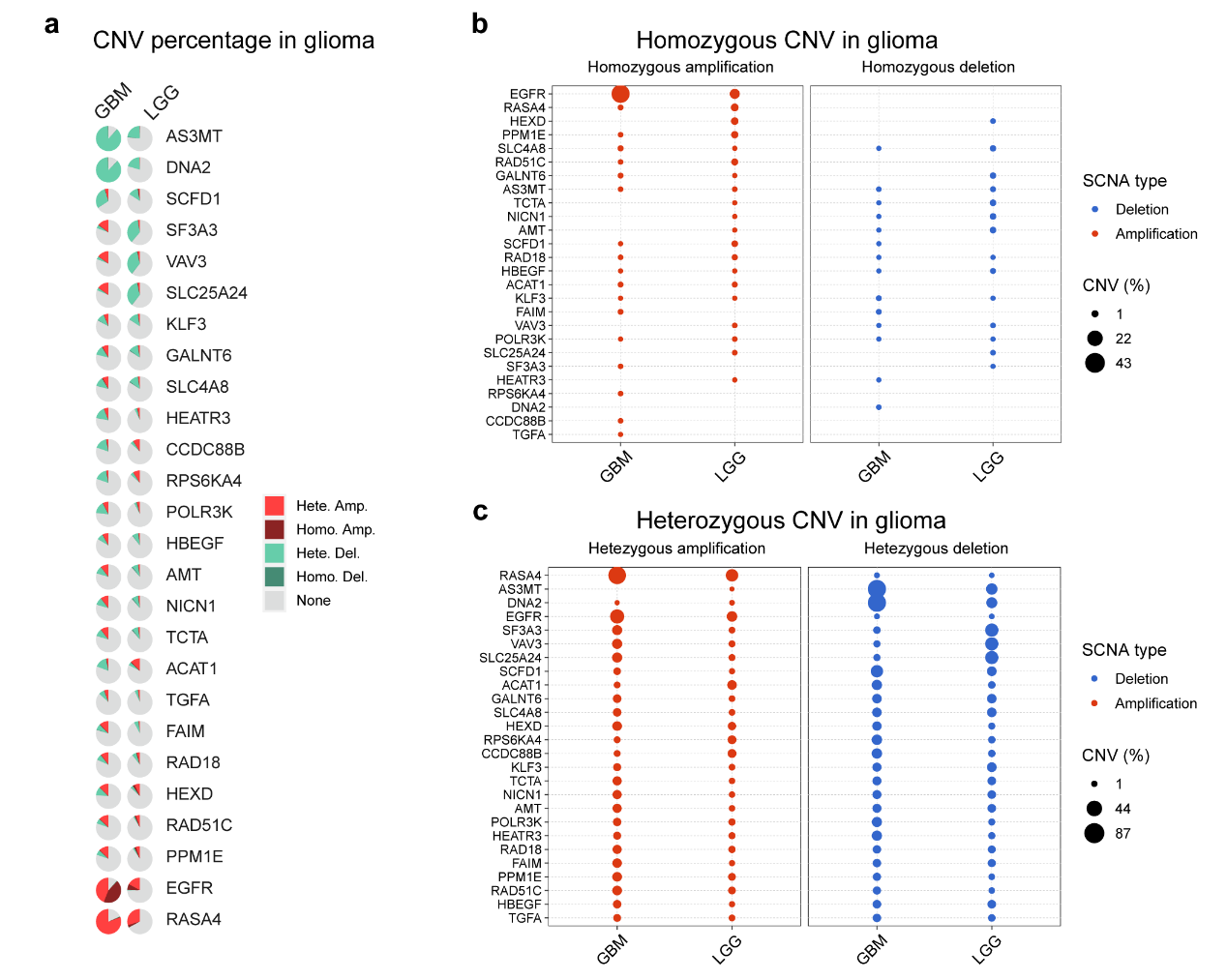
**

Supplementary Fig. 3 | The (**a**) KEGG, (**b**) GWAS Catalog library enrichment analysis and (**c**) PPI network constructed via GeneMANIA database

**
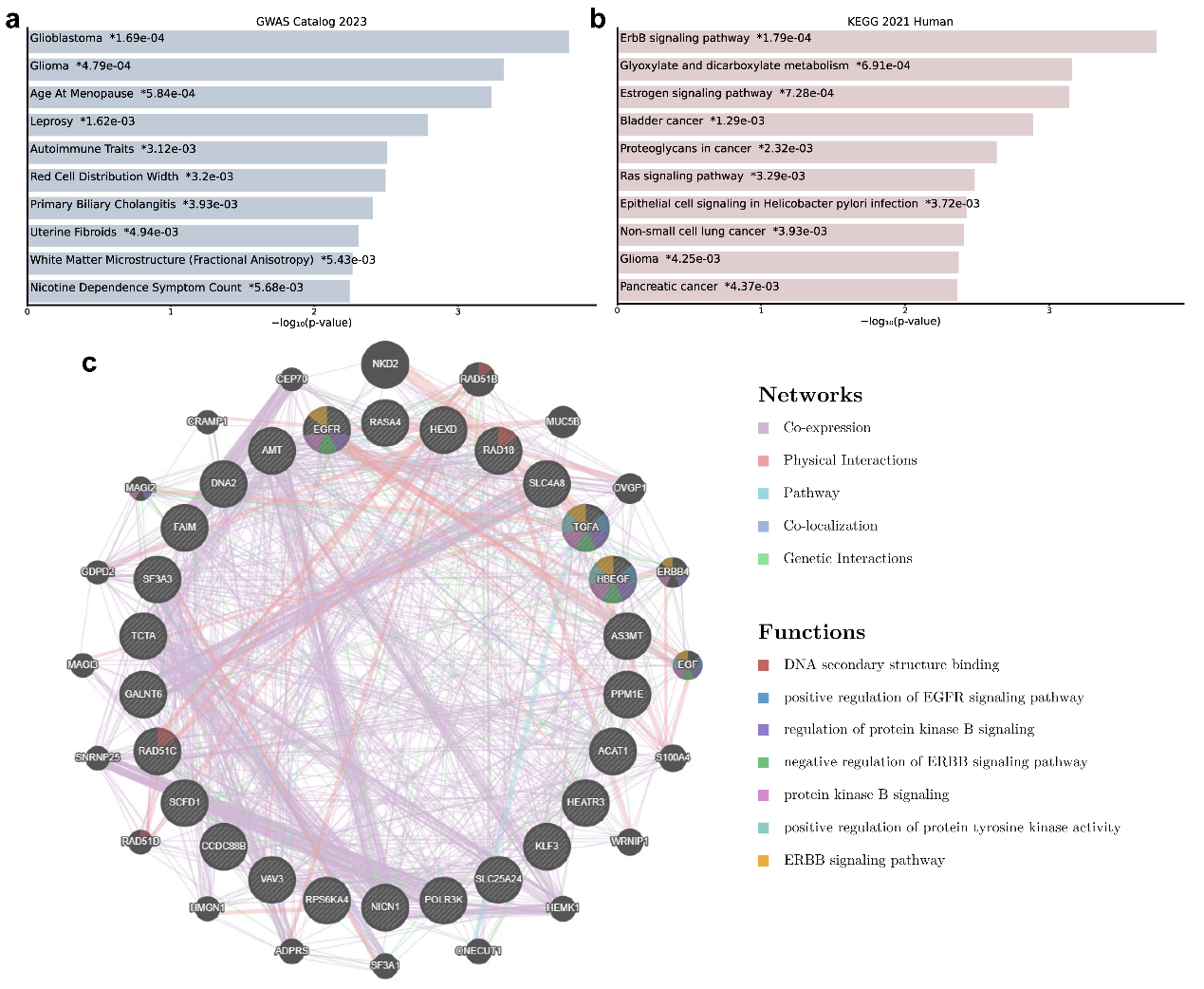
**

Supplementary Fig. 4 | Sex-based differences in gene expression across various types of gliomas (**a-e**) and brain cell types (**f-k**). Significant differences of P < 0.05 are highlighted in red color.


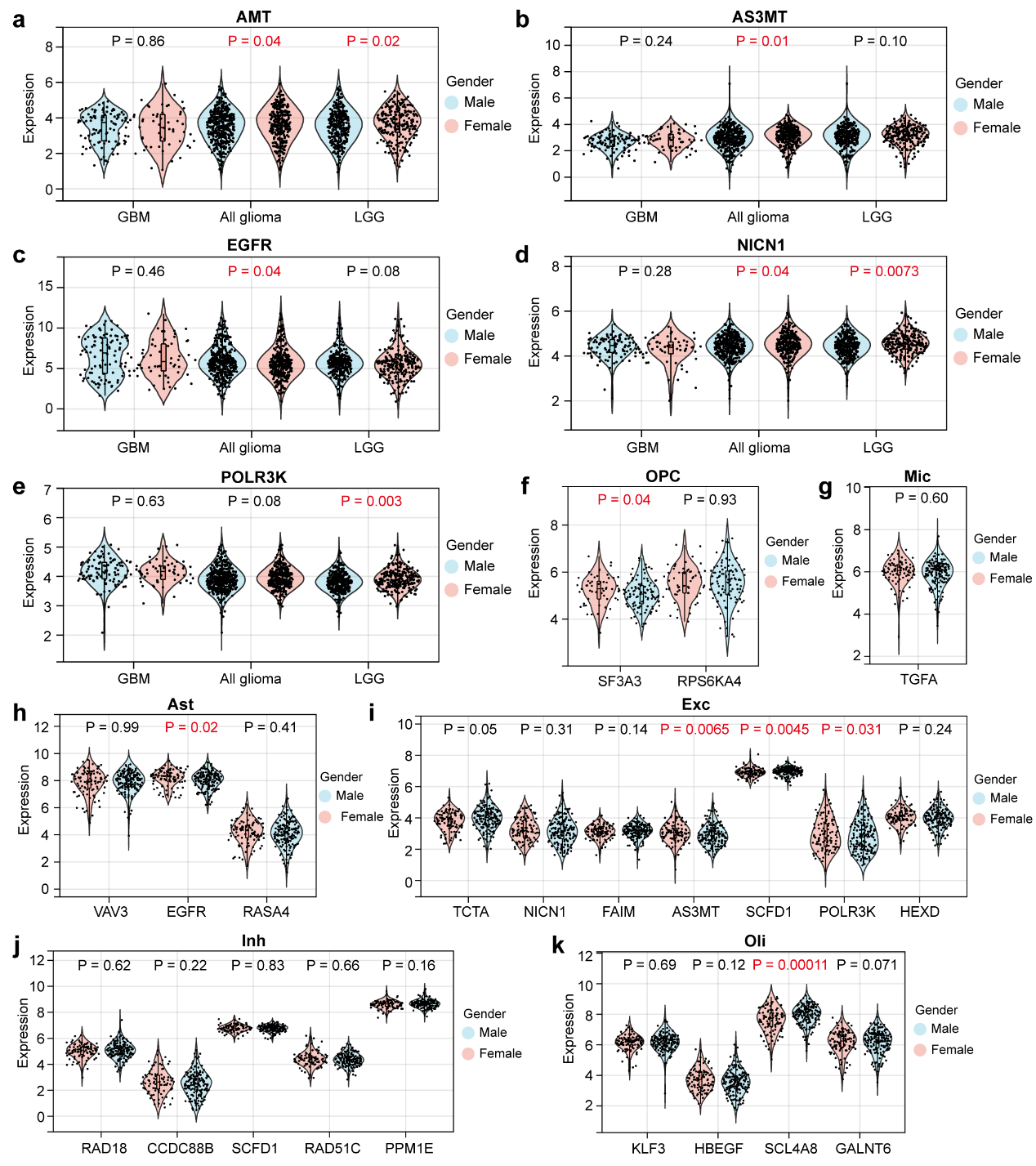


Supplementary Fig. 5 | Representative images of multiplex immunofluorescence for the expression of GFAP (*green*) and DNA2 *(red*) /EGFR (*red*) /VAV3 (*red*) in glioma samples. Nuclei were stained with DAPI (*blue*). Scale bar = 50 μm.

**
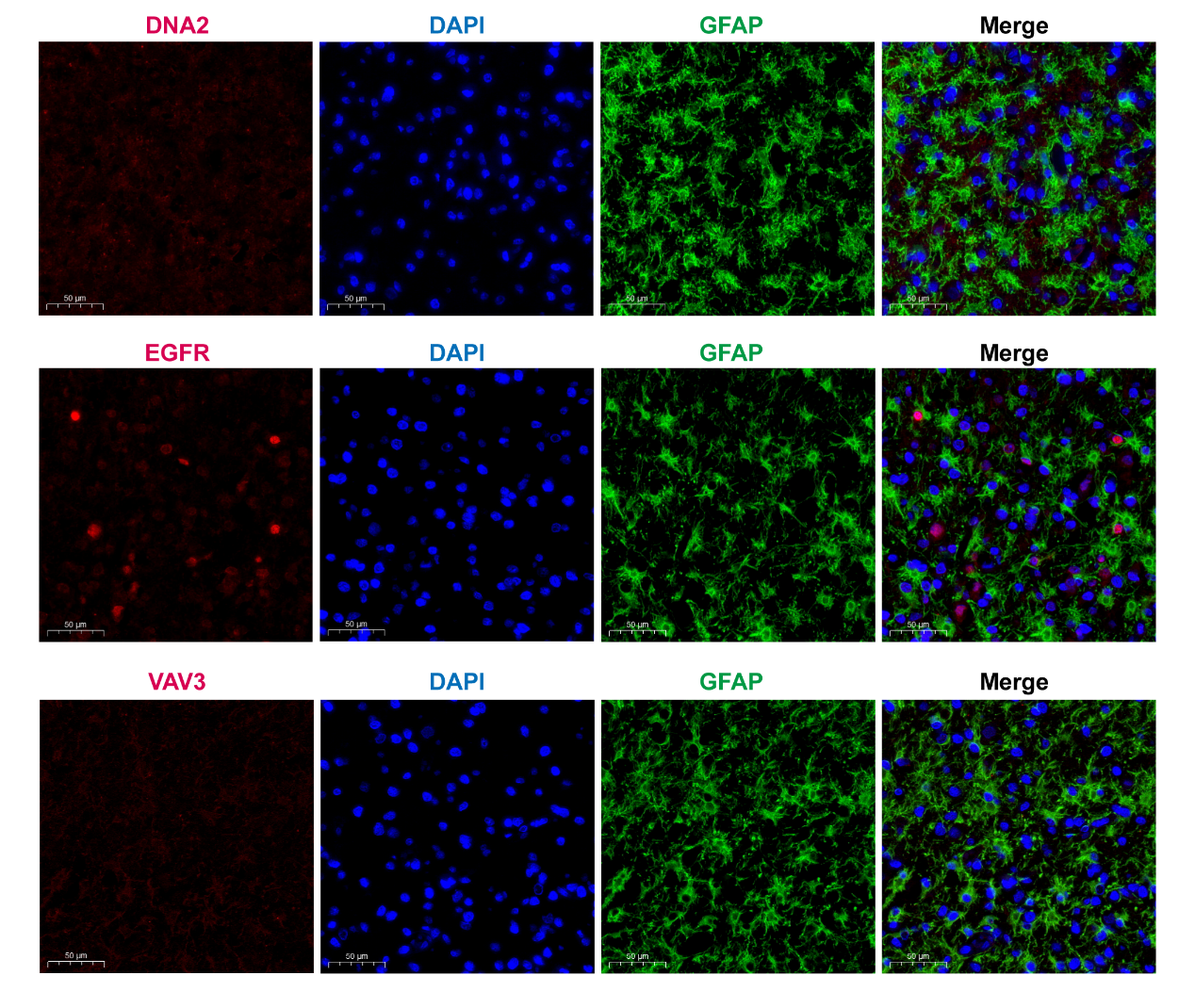
**

Supplementary Fig. 6 | **Representative immunohistochemistry (IHC) staining results for proteins in the cerebral cortex**. Each image displays the staining scores, intensity scores, and fraction of cells with expression of corresponding proteins.


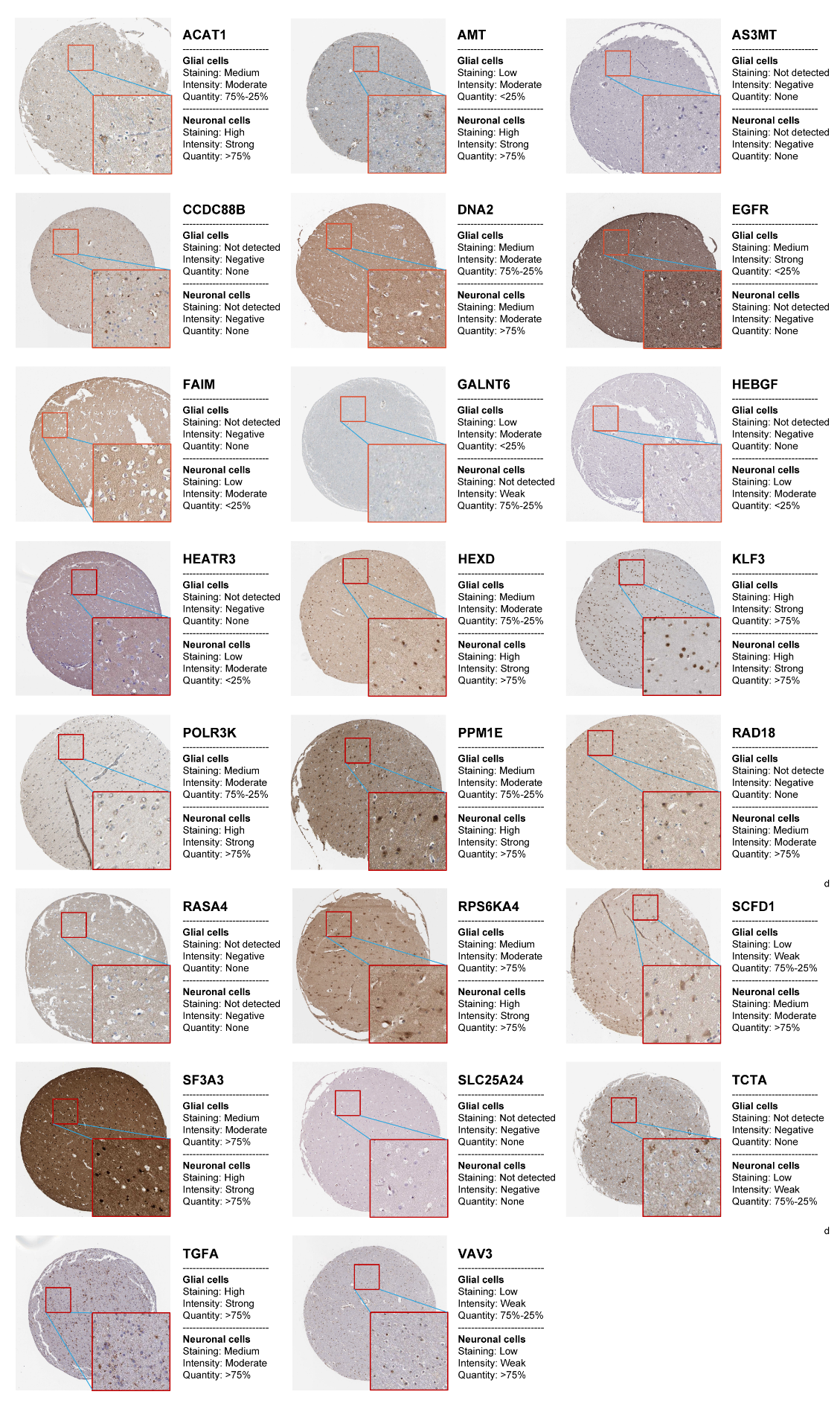


Supplementary Fig. 7 | Protein’s subcellular expression and distribution images of immunofluorescence in glioma cell lines (U-251MG).


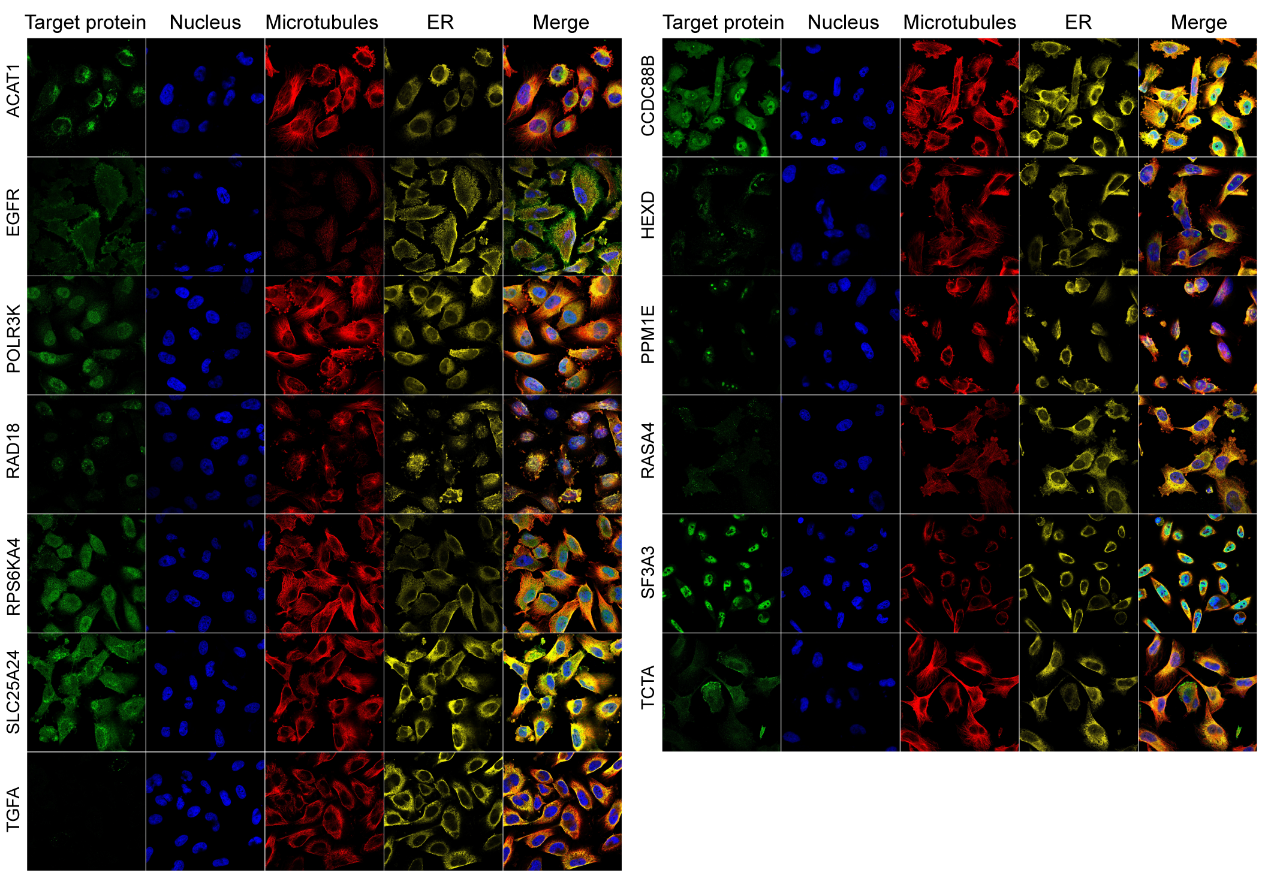


Supplementary Fig. 8 | **Results of drug sensitivity analysis**. **a,b,** Bubble plots summarising the correlations between genes and drugs from (**a**) GDSC and (**b**) CTRP. Drug-gene pairs with an absolute correlation coefficient >0.1 and FDR<0.05 were remained (up to 30 drugs).

**
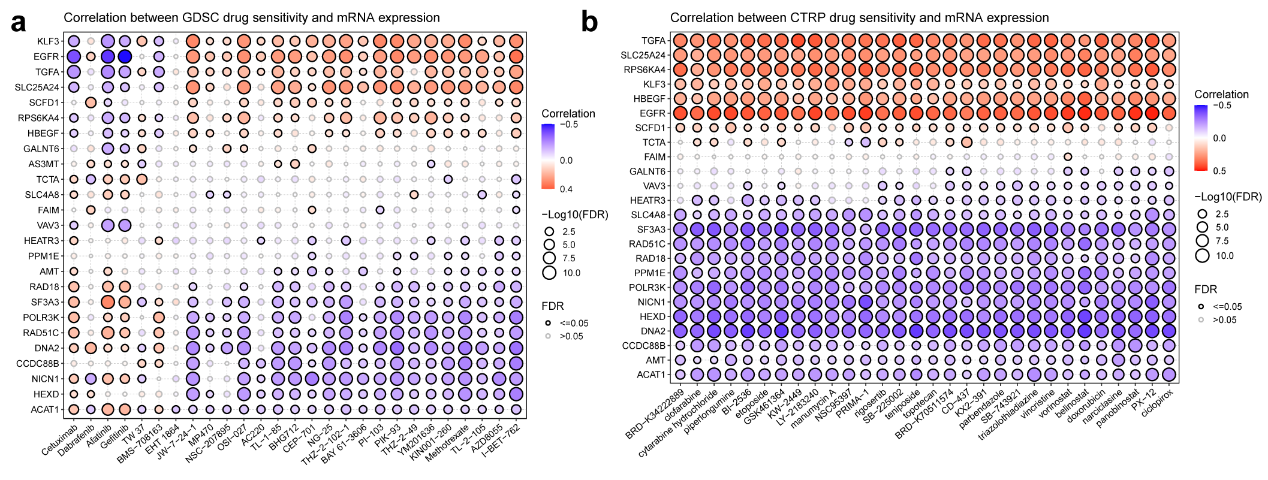
**
